# Optimal risk-assessment scheduling for primary prevention of cardiovascular disease

**DOI:** 10.1093/jrsssa/qnae086

**Published:** 2024-09-17

**Authors:** Francesca Gasperoni, Christopher H Jackson, Angela M Wood, Michael J Sweeting, Paul J Newcombe, David Stevens, Jessica K Barrett

**Affiliations:** MRC Biostatistics Unit, University of Cambridge, Cambridge, UK; MRC Biostatistics Unit, University of Cambridge, Cambridge, UK; Cardiovascular Epidemiology Unit/Department of Public Health and Primary Care, Victor Phillip Dahdaleh Heart and Lung Research Institute, University of Cambridge, Cambridge, UK; Health Data Research UK, London, UK; Department of Health Sciences, University of Leicester, Leicester, UK; MRC Biostatistics Unit, University of Cambridge, Cambridge, UK; Liverpool Centre for Cardiovascular Science, University of Liverpool, Liverpool Heart & Chest Hospital, Liverpool, UK; Department of Cardiovascular and Metabolic Medicine, Institute of Life Course and Medical Sciences, University of Liverpool, Liverpool, UK; MRC Biostatistics Unit, University of Cambridge, Cambridge, UK

**Keywords:** cardiovascular disease, net benefit, optimal risk-assessment, statins initiation, 2-stage landmarking model

## Abstract

In this work, we introduce a personalized and age-specific net benefit function, composed of benefits and costs, to recommend optimal timing of risk assessments for cardiovascular disease (CVD) prevention. We extend the 2-stage landmarking model to estimate patient-specific CVD risk profiles, adjusting for time-varying covariates. We apply our model to data from the Clinical Practice Research Datalink, comprising primary care electronic health records from the UK. We find that people at lower risk could be recommended an optimal risk-assessment interval of 5 years or more. Time-varying risk factors are required to discriminate between more frequent schedules for high-risk people.

## 1 Introduction

The World Health Organization identified cardiovascular disease (CVD) as the leading cause of morbidity and mortality across the world, with 17.9 million deaths from CVD in 2016 (31% of all global deaths, WHO, 2017). The prescription of statins and other lipid-lowering medication is recognized as the most common primary prevention strategy for CVD (Reiner, 2013) with the UK National Institute for Health and Care Excellence (NICE, 2014) guidelines recommending offering atorvastatin 20 mg to people who have a 10% or greater 10-year risk of developing CVD. According to UK guidelines, 10-year CVD risk is recommended to be computed through the QRISK2 assessment tool (Hippisley-Cox et al., 2008) every 5 years from age 40 for both men and women, as part of the NHS Health Check. However, there is no universal agreement on the best risk-assessment strategy (Arnett et al., 2019; Lalor et al., 2012; Piepoli et al., 2016; Pylypchuk et al., 2018). In particular, identifying the optimal CVD risk-assessment frequency is an open problem, as recognized by Piepoli et al. (2016): ‘[repeating CVD risk-assessment occasionally], such as every 5 years, is recommended, but there are no data to guide this interval’. Assessing high-risk individuals too infrequently may delay preventative interventions such as statins initiation, while too frequent assessment of low-risk individuals may incur unnecessary healthcare costs and inconvenience for the individual (Chiolero & Anker, 2019).

The problem of optimal timing for risk assessment is crucial in preventive medicine and it is widely studied in cancer screening (Bibbins-Domingo et al., 2016; Ito et al., 2019; Shieh et al., 2017), but is much less investigated in CVD risk assessment (Lindbohm et al., 2019; Selvarajah et al., 2013). The optimal risk-assessment schedule is often identified via the maximization of a utility function (Rizopoulos et al., 2016; Sweeting, 2017) or via the minimization of a cost function (Bebu & Lachin, 2018). A third option is represented by the net benefit (NB) function defined as the difference between benefits and costs (Gray et al., 2011). These functions are tailored to the specific disease of interest, as they are composed of quantities that are considered discriminatory for that particular condition. Elements evaluated for building these functions might include: the quality-adjusted life years (QALYs) gained, the expected life years gained, the cost associated with a risk-assessment, the expected number of risk assessments, and the undetected time spent with an undiagnosed condition. Furthermore, the optimal risk-assessment schedule could depend on the *stage* of the disease of interest, as for Bebu and Lachin (2018), or on the patient-specific *risk level* of developing the disease, as for Lindbohm et al. (2019). To deal with the dynamic nature of the problem, multi-state models (Bebu & Lachin, 2018; Lindbohm et al., 2019) and joint models (Rizopoulos et al., 2016; Sweeting, 2017) have been investigated. But only a few authors have provided personalized recommendations for the next screening (Bebu & Lachin, 2018; Rizopoulos et al., 2016; Sweeting, 2017).

In this work, we introduce a *personalized* and *age-specific* monitoring schedule that aims to provide an optimal balance between benefits and costs associated with statins initiation. Our recommendations are based on the evidence obtained from large-scale electronic health records (EHR) data. Considering the size of the data and its complexity (i.e. sparse repeated measurements and missing values), joint models and multi-state models would be computationally unfeasible. Instead, we exploit the landmarking framework described by Van Houwelingen and Putter (2011) and at each specific landmark age, we maximize a person-specific NB function. The elements that characterize the net benefit functions are: the CVD-free life years gained over a 10-year time horizon (as benefit); the expected number of visits, the cost associated with a CVD event, the cost of statin consumption (as costs). The idea of considering CVD-free life years and cost of statins for defining a net benefit function was proposed by Rapsomaniki et al. (2012) in a different context (they proposed the NB as an alternative measure for comparing different risk prediction models). A key element in the proposed NB function is the statin initiation time for each person, at each landmark. In order to estimate the statin initiation, we have to define a dynamic CVD risk profile for each person at each landmark. The risk profile is estimated by extending the two-stage landmarking approach by Paige et al. (2018). Specifically, we extend the first stage, by providing not only best linear unbiased current predictions through a linear mixed effect model with random intercept and slope, but also best linear unbiased future predictions of longitudinal risk factors for CVD onset. Exploiting future predictions enables better informed risk-assessment strategies compared to those based only on current risk factors. In the second stage, we estimate the dynamic CVD risk profile by fitting Cox models, adjusting for time-fixed risk factors (recorded at baseline) and time-varying risk factors (known at each landmark age or estimated in the previous stage).

The paper is organized as follows. The motivating dataset is described in Section 2. The proposed model and method is presented in Section 3. The results obtained for men and women separately are shown in Section 4. The final discussion is reported in Section 5.

## 2 Motivating data

Our motivating dataset is derived from the Clinical Practice Research Datalink (CPRD), which covers approximately 6.9% of the UK population and is representative in terms of age and gender (Herrett et al., 2015). This dataset is linked to secondary care admissions from Hospital Episode Statistics (HES), and national mortality records from the Office for National Statistics (ONS) (Herrett et al., 2015). The linked dataset is composed of 2,610,264 patients and 39,189,729 measurements (i.e. body mass index (BMI), high lipoprotein cholesterol, systolic blood pressure (SBP), smoking status, total cholesterol).

We exclude those people with prevalent CVD or statin treatment before study entry. We also exclude individuals who had no measurements of any of BMI , SBP, total cholesterol, high density lipoprotein (HDL) cholesterol, or smoking status between study entry and study exit dates.

We include two different types of risk factors: time-fixed (recorded at baseline and fixed over time) and time-varying (recorded multiple times and may vary over time). We include the Townsend deprivation index as a categorical time-fixed risk factor with 20 levels. Time-varying risk factors comprise: (1) binary risk factors that are assumed zero until the first available health record indicates otherwise and thereafter assumed equal to one (statin consumption index, blood pressure medication index, diagnoses of diabetes, renal disease, depression, migraine, severe mental illness, rheumatoid arthritis, and atrial fibrillation) which we will term medication/disease risk factors, and (2) longitudinal risk factors that can change multiple times: BMI, SBP, total cholesterol, HDL cholesterol (all continuous), and smoking status (current smoker or not) which we will term longitudinal risk factors because they will be modelled using a multivariate linear mixed effects model. CVD is defined as any of the following: acute myocardial infarction, stroke, angina or transient ischemic attack, in line with the definition used in the QRISK3 CVD risk score (Hippisley-Cox et al., 2017).

A total of 1,971,002 individuals (914,951 men and 1,056,051 women) from 406 GP practices were included in the study. We randomly allocated two-thirds of practices (263 practices with 1,337,380 individuals) to the derivation cohort dataset and one-third of practices (135 practices with 633,622 individuals) to the validation cohort dataset. Further details about risk factors and outcome definitions and cohort selection are reported in Section 1 of the online supplementary material.

## 3 Models and methods

We introduce and optimize an NB function in which we account for both benefit and costs associated with risk assessments and statins initiation. The NB function is defined as a *age* and *person-specific* function, whose optimization leads to the identification of a personalized risk-assessment schedule for primary prevention of CVD, at each *age* of interest. We define the ages of interest as our *landmark ages*, $L_{a}=\{40,45,50,\ldots ,80\}$. This choice mimics the current visit schedule recommended by NICE (2014). At each landmark age, we select those people in the derivation set who have not been diagnosed with CVD, are still alive and have not yet received statins, defined as the *landmark cohort*. Each landmark age represents the *time origin* for the NB evaluation, while $L_{a}+10$ represents the *time horizon* (years is the scale), i.e. we consider a potential CVD risk-assessment frequency from every year to every 10 years.

A key point in the model definition is statins initiation, assumed to happen at the first risk assessment scheduled after their 5-year CVD risk exceeds the 5% threshold. Following van Staa et al. (2013), we evaluate 5-year CVD risk instead of 10-year CVD risk due to lack of follow-up. The expected time of crossing the 5% threshold is landmark *age* and *person-specific*, and it is denoted as $t_{i,L_{a}}^{*}$ for a specific person *i*. Statins initiation has a positive impact on lengthening the CVD-free life years (Ferket et al., 2012) and it has been proved to reduce the risk of a CVD event by about 20% as reported by a previous meta-analysis of statin trials (Unit Epidemiological Studies, 2005). Then, we model this effect via the hazard ratio, *θ*, that is set equal to 0.8. Further details on the definition of CVD-free life years are given in Section 3.2.

All analyses are run separately on men and women in the derivation set, since incidence of CVD is substantially higher in men than women.

### 3.1 Net benefit

For each person *i* at $L_{a}$, we define the optimal risk-assessment strategy, $\tau_{i,L_{a}}^{opt}$, as that which maximizes the net benefit function among a set of *F* risk-assessment strategies of interest ($\tau^{f}\in \{\tau^{1},\tau^{2},\ldots ,\tau^{F}\}$) as in equation (1).


(1) $$\tau_{i,L_{a}}^{opt}=\underset{\;f\in \{1,\ldots ,F\}}{\text{arg max}}\;NB_{i,L_{a}}(\tau^{f}).$$

The risk-assessment schedule, $\tau^{f}$, is a vector of visit times $\tau^{f}=\{\tau_{1}^{f},\tau_{2}^{f},\ldots ,\tau_{V}^{f}\}$, characterized by *f*, the time between two visits (i.e. f=1 stands for yearly evaluation). Note that the visit times are all fixed in advance and are defined by a specific time interval *f* and $\tau_{1}^{f}$ is always equal to the origin time, $L_{a}$, while $\tau_{V}^{f}\leq L_{a}+10$ (e.g. for f=1 visit times are $L_{a},L_{a}+1,L_{a}+2,\ldots ,L_{a}+10$). The risk-assessment scheduled after person *i* is expected to have a 5-year CVD risk higher than 5%, $t_{i,L_{a}}^{*}$ at landmark age $L_{a}$, is denoted as $\tau_{k_{i,L_{a}}^{*}}^{f}$. To avoid overly heavy notation, we drop the superscript *f* in the remaining part of this section.

$NB_{i,L_{a}}(\tau )$ is defined in monetary terms, by converting health outcomes to the scale of costs, and subtracting the actual costs of health service usage, if required. Health outcomes are measured as expected QALYs, over a maximum time of 10 years, and can be converted to expected costs by multiplying the amount *λ* that a decision-maker is willing to pay for 1 year of full health. We assume that *λ* ranges from £20,000/year to £30,000/year (NICE , 2014). The expected costs are composed of all costs associated with the expected CVD-free life years of a person (up to a maximum of 10 years), comprising the yearly cost of statins taken after $\tau_{k_{i,L_{a}}^{*}}$ and the expected costs of risk-assessment visits.

First, the $NB_{i,L_{a}}(\tau )$ is defined in equation (2).


(2) $$NB_{i,L_{a}}(\tau )=QALY(\tau )\cdot \lambda -cost(\tau ).$$

QALY(τ) is based on the following elements:

-
$EFLY_{NS}(\tau_{k_{i,L_{a}}^{*}})$ : Time *before statin initiation* spent free of CVD, or event-free life years (EFLY) without statins. This time can be computed as the integral of the probability of not developing CVD, with no statins initiation, between time origin and $\tau_{k_{i,L_{a}}^{*}}$.
-
$EFLY_{S}(\tau_{k_{i,L_{a}}^{*}})$ : Time *after statin initiation* spent free of CVD or EFLY with statins. This time can be computed as the integral of the probability of not developing CVD, after statins initiation, between $\tau_{k_{i,L_{a}}^{*}}$ and time horizon.

We assume that $EFLY_{NS}(\tau_{k_{i,L_{a}}^{*}})$ is associated with a utility equal to 1 (full health), while $EFLY_{S}(\tau_{k_{i,L_{a}}^{*}})$ is associated with a disutility $u_{s}$. Statins are considered to be very low risk drugs, associated with a utility reduction from 0 to 0.003, given to *pill burden* (Kong & Zhang, 2018). This means that $u_{s}\in [0.997,1]$. Refer to equation (3) for the extended definition of QALY(τ).


(3) $$QALY(\tau )=EFLY_{NS}(\tau_{k_{i,L_{a}}^{*}})+u_{s}\cdot EFLY_{S}(\tau_{k_{i,L_{a}}^{*}}).$$

The expected costs associated with a predefined risk-assessment strategy τ are composed of the yearly cost of statins, $c_{s}$ [£/year], taken for $EFLY_{S}(\tau_{k_{i,L_{a}}^{*}})$ years, the expected costs of visits, defined as the cost of a single visit $c_{\nu }$ [£/visit] multiplied by the expected number of visits, $E_{\tau }[N_{i}]$


(4) $$cost(\tau )=c_{s}\cdot EFLY_{S}(\tau_{k_{i,L_{a}}^{*}})+c_{\nu }\cdot E_{\tau }[N_{i}].$$

**Figure 1.**
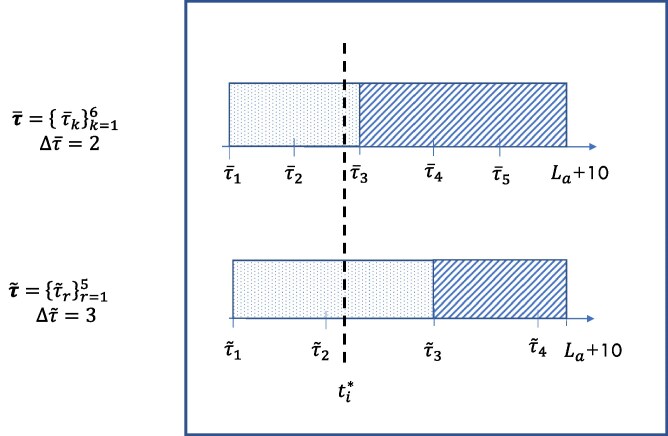
Example figure for describing the procedure for computing $k_{i}^{*}$, $\tau_{k_{i}^{*}}$, given $t_{i}^{*}$ and two specific risk-assessment strategies. In the upper part of the figure, we consider risk-assessment schedule $\bar{\tau }$ (visits every 2 years), while in the lower part, we consider another risk-assessment schedule $\tilde{\tau }$ (visits every 3 years). This person is expected to cross the 5% threshold at $t_{i}^{*}$, which implies that this person is going to start taking statins at the third visit (${\bar{k}}_{i}^{*}={\tilde{k}}_{i}^{*}=3$) in both cases ($E_{\bar{\tau }}[N_{i}]=E_{\tilde{\tau }}[N_{i}]=3$). However, ${\bar{\tau }}_{3}$ and ${\tilde{\tau }}_{3}$ are different, ${\bar{\tau }}_{3}<{\tilde{\tau }}_{3}$, which means that time spent under statins (boxes with diagonal patterns) is longer if we focus on $\bar{\tau }$ (risk-assessment every 2 years). The statin-free time is represented through boxes with dotted patterns.

The cost of statins per year of life, $c_{s}$ ranges from £4.3/year to £321.2/year, assuming a daily dose of 20 mg of Atorvastatin (Joint Formulary Committee, 2020). The cost of a single visit, $c_{\nu }$ is assumed to be £18.39/visit (Kypridemos et al., 2018). To estimate $E_{\tau }[N_{i}]$, we assume that the CVD risk assessments are performed up to time $\tau_{k_{i}^{*}}$ (i.e. no more visits after statins initiation). In Figure 1, we represent an illustrative example to show how the expected number of visits is computed for two different risk-assessment schedules ($\bar{\tau }$ in the top row and $\tilde{\tau }$ in the bottom row) for a generic person whose 5-year CVD risk exceeds the 5% threshold at $t_{i}^{*}$ (dashed black line). According to both risk-assessment schedules, person *i* should start taking statins from the third visit, which means ${\bar{k}}_{i}^{*}={\tilde{k}}_{i}^{*}=3$ and $E_{\bar{\tau }}[N_{i}]=E_{\tilde{\tau }}[N_{i}]=3$. If a person never crosses the 5% threshold, they will never start taking statins and the expected number of visits including the baseline visit is $E_{\tau }[N_{i}]=1+10/\Delta \tau$, where Δτ is the time between visits according to visit schedule τ. From Figure 1, the expected number of visits for a person whose 5-year CVD risk never crosses the 5% threshold are 6, according to $\bar{\tau }$ and 4.33, according to $\tilde{\tau }$.

By combining the equations (2) and (4), we are able to define the net benefit function for the *i*th person at landmark age $L_{a}$, associated with a specific risk-assessment schedule τ.

To compute equation (2), we estimate the $EFLY_{NS}(\tau_{k_{i,L_{a}}^{*}})$, $EFLY_{S}(\tau_{k_{i,L_{a}}^{*}}),$ and $E_{\tau }[N_{i}]$ . The event-free life years are estimated through a 2-stage landmarking approach where the event of interest is a CVD diagnosis between $L_{a}$ and $L_{a}+10$ (see Section 3.2). Considering the definition of $\tau_{k_{i,L_{a}}^{*}}$ as the first visit after $t_{i,L_{a}}^{*}$, the problem collapses to the prediction of $t_{i,L_{a}}^{*}$. We provide an extended 2-stage landmarking model in Section 3.3 to estimate $t_{i,L_{a}}^{*}$. We set the values of *λ*, $u_{s}$, $c_{s}$, $c_{\nu }$ in Section 4 and run a sensitivity analysis to assess the robustness of our analysis with respect to the variability of these parameters (Section 5 of the online supplementary material).

Two special cases of equation (1) can be identified. The first one is when the 5-year CVD risk of a person is not expected to cross the 5% threshold in the time-window of interest $\left[L_{a},L_{a}+10\right]$, which means $\tau_{k_{i,L_{a}}^{*}}\geq L_{a}+10$. In this case, equation (1) is driven by the term related to the expected number of visits. The optimal risk-assessment strategy is therefore the one associated with the lowest expected number of visits $E_{\tau }[N_{i}]$. The second case is when the 5-year CVD risk is predicted higher than the 5% threshold at $L_{a}$, which means $\tau_{k_{i,L_{a}}^{*}}=L_{a}$. We are not interested in evaluating this case, as these people should initiate statins already at $L_{a}$.

### 3.2 Two-stage landmarking approach for CVD risk prediction

In this section, we describe how we apply the original two-stage landmarking model proposed by Paige et al. (2018), while in Section 3.3 we explain how we extend the 2-stage landmarking model to provide future predictions at the times of interest ($L_{a}+1,L_{a}+2,\ldots ,L_{a}+10$) conditional on the information available at landmark age $L_{a}$.

In the first stage, at each landmark age $L_{a}\in \{40,45,\ldots ,80\}$, we fit a linear mixed effect model (LMEM) with random intercepts and slopes to all individuals from the derivation dataset who are in the *landmark cohort*. The outcomes of interest are the longitudinal risk factors that can change multiple times. Let $smoke_{ij}$, $SBP_{ij}$, $TCHOL_{ij}$, $HDL_{ij}$, $BMI_{ij}$, $BPM_{ij}$, $statin_{ij}$, and $age_{ij}$ denote the repeated measurements of smoking status, systolic blood pressure, total cholesterol, HDL cholesterol, body mass index, history of blood pressure-lowering medication, statin prescription, and age for individual *i*, $i\in \{1,\ldots ,N_{L_{a}}\}$, recorded at visit *j*, $j\in \{1,\ldots ,J_{i}\}$, where $N_{L_{a}}$ is the landmark cohort size. In order to get the most precise estimates of the model regression parameters, we include not only past measurements taken prior to the landmark age, but also future measurements (Paige et al., 2018). The LMEM is defined as


(5) $$\begin{array}{c} smoke_{ij}=\beta_{10}+\beta_{11}age_{ij}+u_{10i}+u_{11i}age_{ij}+\varepsilon_{ij}\\ HDL_{ij}=\beta_{20}+\beta_{21}age_{ij}+u_{20i}+u_{21i}age_{ij}+\varepsilon_{ij}\\ SBP_{ij}=\beta_{30}+\beta_{31}age_{ij}+\beta_{32}BPM_{ij}+u_{30i}+u_{31i}age_{ij}+\varepsilon_{ij}\\ TCHOL_{ij}=\beta_{40}+\beta_{41}age_{ij}+\beta_{42}statin_{ij}+u_{40i}+u_{41i}age_{ij}+\varepsilon_{ij}\\ BMI_{ij}=\beta_{50}+\beta_{51}age_{ij}+u_{50i}+u_{51i}age_{ij}+\varepsilon_{ij} \end{array}$$

where $\left(\begin{array}{c} u_{0i}\\ u_{1i} \end{array}\right)∼MVN(0,\Sigma )$ and *Σ* is a full matrix; $\varepsilon_{ij}∼MVN(0,\sigma_{e}I)$ and *I* is the identity matrix. Here $\beta_{0}=(\beta_{10},\beta_{20},\beta_{30},\beta_{40},\beta_{50})$ represents fixed intercepts for each risk factor, $\beta_{1}=(\beta_{11},\beta_{21},\beta_{31},\beta_{41},\beta_{51})$ represents fixed slope for each risk factor. $\beta_{32}$ represents an adjustment factor in systolic blood pressure levels for those subjects under blood-pressure lowering medication at the time the measurement was taken. $\beta_{42}$ is the regression parameter that represents the effect of statin prescription on total cholesterol. $u_{0i}$ and $u_{1i}$ are vectors of risk factor-specific random intercepts and random slopes respectively and are correlated between risk factors. Finally, $\varepsilon_{ij}$ represents uncorrelated residual errors for each risk factor.

Our model assumes that all risk factors jointly follow a multivariate normal distribution, which is plausible for BMI, SBP, total cholesterol, HDL cholesterol but less plausible for smoking status which is a dichotomous variable. However, inference based from the multivariate normal distribution may often be reasonable even if multivariate normality does not hold (Schafer, 1997).

We complete the first stage of the two-stage landmarking approach by predicting *current longitudinal risk factor values* using the best linear unbiased predictors (BLUPs) for each person *i* of the landmark cohort, at time $L_{a}$ (denoted as ${\hat{SBP}}_{iL_{a}}$, $\hat{TCHOL_{iL_{a}}}$, ${\hat{smoke}}_{iL_{a}}$, ${\hat{HDL}}_{iL_{a}}$, and ${\hat{BMI}}_{iL_{a}}$). Importantly, we only take advantage of *past observations* for computing the BLUPs because the prediction of the longitudinal CVD risk factors should not depend on future information. The prediction of the BLUPs therefore mirrors the prediction as it would be carried out for a new individual who we have only observed up to the landmark age.

In the second stage of the landmarking approach, we fit a Cox proportional hazard model at each landmark age. The event of interest is the time to CVD diagnosis over the next 10 years (people diagnosed with CVD after the time horizon $L_{a}+10$ are censored). The risk factors included in the Cox proportional hazard model at time $L_{a}$, are of two types: *known* at landmark age or *estimated* at landmark age. The risk factors *known* at $L_{a}$ are diabetes, blood pressure medication, renal disease, depression, migraine, severe mental illness, rheumatoid arthritis, atrial fibrillation diagnosis, and Townsend deprivation score. These risk factors are assumed known at the landmark age $L_{a}$ and are assumed to be constant over time from the landmark age. We denote these risk factor values for person *i* as $x_{i,L_{a}}$. The risk factors *estimated* at $L_{a}$ are BMI, SBP, total cholesterol, HDL cholesterol, and smoking status. The values included in the Cox model at time $L_{a}$ are the BLUPs *resulting from the first stage*. We refer to these values for person *i* as $x_{i,BLUP}(L_{a})$. We assume the hazard of being diagnosed with CVD, without taking statins, considering $L_{a}$ as the origin time, and conditional on the values of time-fixed and time-varying covariates known or estimated at the landmark age, in equation (6).


(6) $$\lambda^{NS}(t;x_{i}(L_{a}),L_{a})=\lambda_{0}^{NS}(t;L_{a})\cdot \text{exp}\left\{x_{i,L_{a}}^{T}\beta_{L_{a}}+x_{i,BLUP}^{T}(L_{a})\beta_{BLUP}(L_{a})\right\}.$$

Given equation (6), we are able to estimate the probability a person will not be diagnosed with CVD by time t, given they are not on statins, $S^{NS}(t;x_{i}(L_{a}),L_{a})$, that is $\Lambda_{0}(t;L_{a})$  $\text{exp}\{x_{i}(L_{a}{)}^{T}\beta (L_{a})\}$, where $\Lambda_{0}(t;L_{a})$ is the cumulative baseline hazard and $x_{i}(L_{a})$ is the vector of *all* risk factors of a person *i*, measured at $L_{a}$. Following the definition given in the previous section, we can write the $EFLY_{NS}(\tau_{k_{i,L_{a}}^{*}})$ as in equation (7).


(7) $$EFLY_{NS}(\tau_{k_{i,L_{a}}^{*}})=\int_{L_{a}}^{\tau_{k_{i,L_{a}}^{*}}}S^{NS}(t;x_{i}(L_{a}),L_{a})\;dt.$$

Analogously, the $EFLY_{S}(\tau_{k_{i,L_{a}}^{*}})$ is reported in equation (8).


(8) $$EFLY_{S}(\tau_{k_{i,L_{a}}^{*}})=\int_{\tau_{k_{i,L_{a}}^{*}}}^{L_{a}+10}S^{S}(t;x_{i}(L_{a}),L_{a})\;dt$$

where $S^{S}(t;x_{i}(L_{a}),L_{a})$ is the probability of not being diagnosed with CVD after statins initiation and is equal to $S^{NS}(\tau_{k_{i}^{*}};x_{i}(L_{a}))\times (\frac{S^{NS}(t;x_{i}(L_{a}))}{S^{NS}(\tau_{k_{i}^{*}};x_{i}(L_{a}))}{)}^{\theta }$, where *θ* is the hazard ratio derived from a meta-analysis of statins trial and set equal to 0.8 (Unit Epidemiological Studies, 2005). Complete details on the derivation of $S^{S}(t;x_{i}(L_{a}),L_{a})$ can be found in Section 2.1 of the online supplementary material.

### 3.3 Extending the 2-stage model for predicting the 5% crossing time

We introduce the extension to the 2-stage landmarking model, required to provide the 5-year CVD personalized risk profile to predict $t_{i,L_{a}}^{*}$, conditional on the history of the person at landmark age $L_{a}$.

First, we define the *prediction time set* at landmark age $L_{a}$ ($P_{L_{a}}=\{L_{a},L_{a}+1,$  $L_{a}+2,\ldots ,L_{a}+10\}$), as the collection of times at which we want to estimate the 5-year CVD risk after the current landmark age. The whole landmark cohort will not be alive after 1, 2, 3…,10 years and it would not be sensible to predict values for people that died or have been diagnosed with CVD before the time of interest *s*, $s\in P_{L_{a}}$. Therefore, it is necessary to create a *sub-cohort* composed only of those people that are still alive and are not diagnosed with CVD at each prediction time *s*, $s\in P_{L_{a}}$. Using the LMEM (5) fitted to individuals in the landmark cohort in Section 3.2, we are able to compute ${\hat{SBP}}_{is}$, $\hat{TCHOL_{is}}$, ${\hat{smoke}}_{is}$, ${\hat{HDL}}_{is}$, and ${\hat{BMI}}_{is}$ as the BLUPs for each person *i* belonging to the *landmark sub-cohort*, at each time *s* in the prediction time set $P_{L_{a}}$.

Given the BLUPs computed at each time $s\in P_{L_{a}}$, we fit a Cox proportional hazard model at each time *s*, on the landmark sub-cohort. We are interested in 5-year CVD risk prediction, so all events happening later than s+5, $s\in P_{L_{a}}$, are considered as censored at time s+5.

We use a standard Cox proportional hazard model fitted at each prediction time *s*, $s\in P_{L_{a}}$ in equation (9). This equation is identical to equation (6), apart from (i) the origin time *s* ($L_{a}$ in the previous section, here $s\in P_{L_{a}}$); (ii) the BLUPs of SBP, total cholesterol, HDL, BMI, and smoking (here evaluated not just at $L_{a}$, but at each time in $P_{L_{a}}$); (iii) the cohort under analysis is the landmark cohort (composed of $N_{L_{a}}$ individuals) in equation (6), while it is the landmark sub-cohort (composed of $N_{L_{a},s}$ individuals) in equation (9); (iv) the window of interest (10 years and 5 years respectively).


(9) $$\begin{array}{rl} \lambda (t;x_{i}(s),s) & =\lambda_{0}(t;s)\cdot \text{exp}\left\{x_{i,s}^{T}\beta_{s}+x_{i,BLUP}^{T}(s)\beta_{BLUP}(s)\right\}\\ & \;i\in \{1,\ldots ,N_{L_{a},s}\}\;s\in P_{L_{a}}\;s\leq t\leq s+5. \end{array}$$

Given the Nelson–Aalen estimator of the cumulative hazard function $\hat{\Lambda }(t;s)$, s≤t≤s+5, and the estimated regression parameters $\hat{\beta }(s)$, $s\in P_{L_{a}}$, from equation (9) and denoting $x_{i}(s)$ as the vector of all covariates of person *i* measured at time *s* and the BLUPs estimated at time *s*, we are able to estimate the 5-year CVD risk ${\hat{r}}_{i}(s+5;x_{i}(s),s)$ for person *i* as follows:


(10) $$\hat{r}(s+5;x_{i}(s),s)=1-\text{exp}\{-{\hat{\Lambda }}_{0}(s+5;s)\cdot \text{exp}\{x_{i}^{T}(s)\hat{\beta }(s)\}\}\;i=\{1,\ldots ,N_{L_{a},s}\},\;s\in P_{L_{a}}.$$

At each landmark age $L_{a}$, we are able to compute a vector of 5-year CVD risks for each individual *i* in the landmark cohort. The elements of this vector are the 5-year CVD risk ${\hat{r}}_{i}(s+5;x_{i}(s),s)$ estimated at each time $s\in P_{L_{a}}$.

Finally, we predict the time $t_{i,L_{a}}^{*}$ at which the 5-year CVD risk of person *i* exceeds the 5% threshold, by linearly interpolating between the first year we estimate a 5-year CVD risk higher than 5% and the previous year. Note that a person may not cross the risk threshold at all for any *s* in the prediction time set.

We validate all prediction models using the dynamic concordance index (Harrell et al., 1996; Van Houwelingen & Putter, 2011) and the dynamic Brier Score (Graf et al., 1999; Van Houwelingen & Putter, 2011). Validation was performed using the validation dataset to avoid overfitting. See Section 4 of the online supplementary material for more details.

## 4 Results

**Figure 2.**
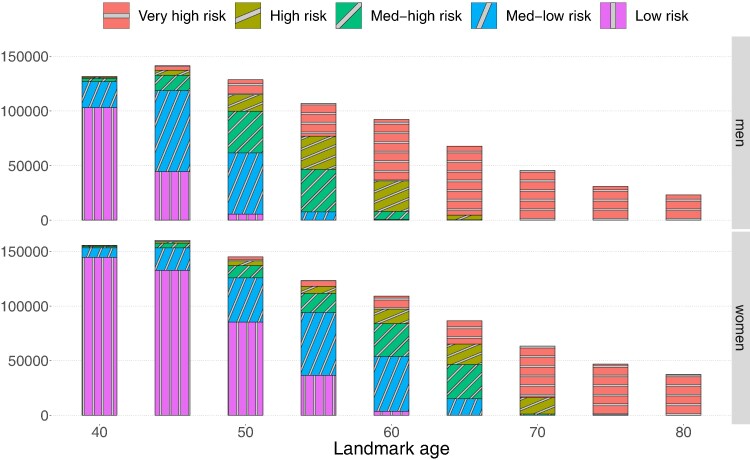
Number of participants in each landmark cohort for men (top row) and women (bottom row) across all landmark ages, in the derivation set. Each colour represents the estimated 5-year CVD risk at the landmark age. This figure appears in colour in the electronic version of this article.

The sizes of the selected landmark cohorts for men (top row) and women (bottom row) are reported in Figure 2. The colours represent a classification of the 5-year CVD risk at different landmark ages, i.e. $\hat{r}(L_{a}+5;x_{i}(L_{a}),L_{a})$ from equation (10). The classification is the following: very high if >5%; high if in the interval (3.75%,5%]; medium high if in the interval (2.5%,3.75%]; medium-low if in the interval (1.25%,2.5%]; low if ≤1.25%. Note that the biggest landmark cohort size is recorded at landmark age 45 for both women and men. This is not anomalous because people can enter the study after age 40 (see Section 2). Moreover, as the landmark ages increases, we observe that the proportion of very high-risk people increases, while the proportion of low-risk people decreases. But note that the sub-cohorts computed at each landmark can only decrease in size. Following the considerations made at the end of Section 3.1, we exclude people at very high risk from our risk-assessment strategy evaluation.

### 4.1 Optimal risk-assessment strategy

In this subsection, we present the optimal risk-assessment strategy resulting from equation (1) for all individuals at each landmark age. We set the parameters of equation (1) as follows: λ=25,000 £/year, $u_{s}=0.997$, $c_{s}=150$ £/year, and $c_{\nu }=18.39$ £/visit. We evaluated equation (1) at F=10 different risk-assessment schedules $\tau^{\;f}$, f∈{1,…,10}. A schedule of risk-assessments every 5 years (f=5) corresponds to the recommendation of the NICE guidelines.

We represent the result of net benefit evaluation at landmark age 40 for women and men in Table 1. We observe that for high-risk individuals more frequent schedules are preferred in general, whereas for people at low or medium-low risk a 10-year schedule appears appropriate. The classification as low risk at the landmark age is a good proxy for recommending a 10-year risk-assessment strategy. However, a range of risk-assessment recommendations may be made for individuals with a higher 5-year CVD risk at the landmark age, due to the extra information provided by the values of specific current and future predicted risk factors for those individuals, that is exploited by our prediction model.

**Table 1.** Optimal risk-assessment strategy for woman and men at landmark age 40 (*f* represents the time in years between two visits)

	*f*	High risk	Med-high risk	Med-low risk	Low risk	
Women	1	78	7	0	0	85 (0.05%)
	2	85	49	5	0	139 (0.09%)
	3	63	59	11	0	133 (0.09%)
	4	6	32	32	0	70 (0.05%)
	5	79	98	0	0	177 (0.11%)
	6	63	10	0	0	73 (0.05%)
	10	109	1,323	8,609	144,419	154,460 (99.56%)
	Total	483 (0.31%)	1,578 (1.02%)	8,657 (5.58%)	144,419 (93.09%)	
Men	1	171	2	0	0	173 (0.13%)
	2	291	91	4	0	386 (0.3%)
	3	251	194	11	0	456 (0.35%)
	4	149	349	259	0	757 (0.58%)
	5	79	786	544	0	1,409 (1.08%)
	6	31	685	849	0	1,565 (1.2%)
	7	15	233	0	0	248 (0.19%)
	10	8	730	22,154	102,895	125,787 (96.18%)
	Total	995 (0.76%)	3,070 (2.35%)	23,821 (18.21%)	102,895 (78.68%)	

*Note*. Women categorized as very high risk are 360 (0.23%) of 155,497, while men at very high risk are 767 (0.58%) of 131,548.

We can observe in Table 1 that the greatest part of both cohorts is categorized as low risk (93.09% women and 78.68% men), while only 483 (0.31%) women and 995 (0.76%) men are labelled as high risk. For almost the whole female cohort (99.56%) at this landmark age, and for 96.18% of the male cohort, undergoing visits every 10 years is found to be the optimal configuration. Focusing on high-risk people, we note that the most recommended risk-assessment strategy is every 1, 2, 3, and 4 years (more evident for men than women). However, there are a few people at high risk whose risk-assessment could be performed every 10 years at landmark age 40 (109 women and 8 men). This is because some individuals are predicted flat trends in their 5-year CVD risk profiles. A focus on the 5-year CVD risk profiles for women labelled as high risk at $L_{a}=40$ is reported in Figure 2 of the online supplementary material.

Furthermore, people classified at low risk at $L_{a}$ are often not expected to initiate statins in the next 10 years. Indeed, looking at Tables 1 and 2 in online supplementary material, we notice that the 5-year CVD risk is not expected to cross the 5% threshold for 143,891 of the 144,419 women labelled as low risk at landmark age 40 and for 100,271 of the 102,895 men labelled as low risk at landmark age 40.

**Figure 3.**
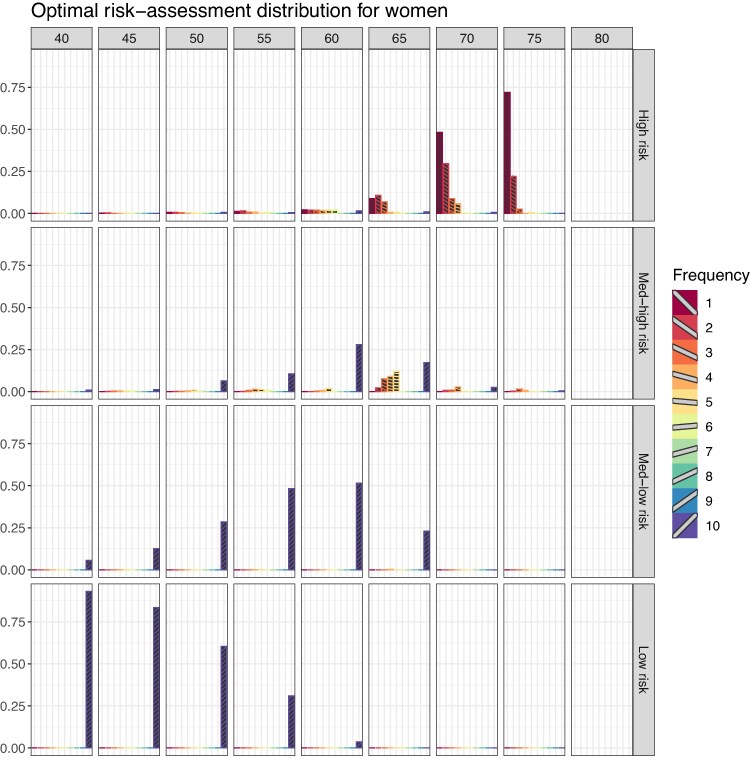
Proportions of optimal risk-assessment schedule per each landmark age, for women. This figure appears in colour in the electronic version of this article.

**Figure 4.**
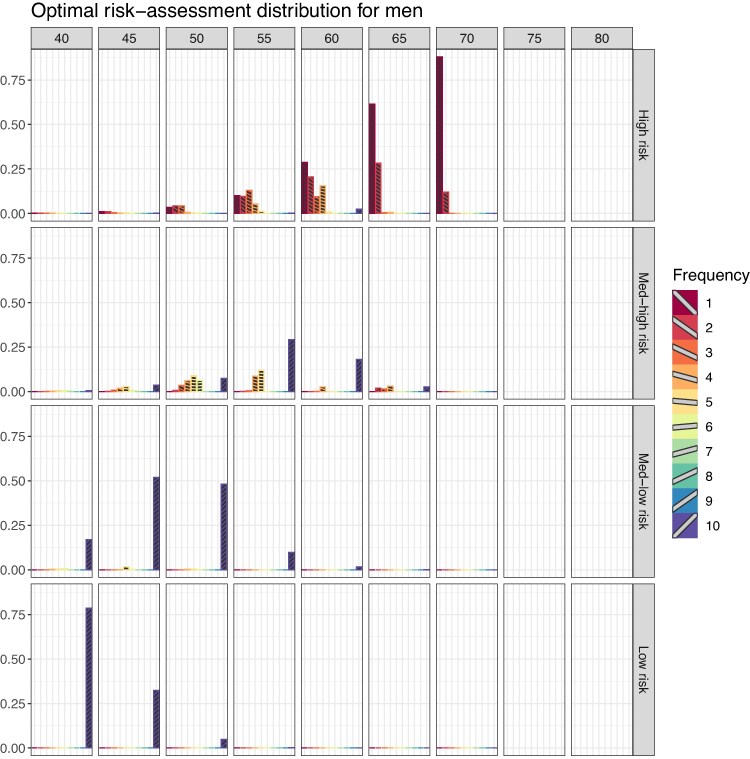
Proportions of optimal risk-assessment schedule per each landmark age, for men. This figure appears in colour in the electronic version of this article.

An overview of the results for women and men across all landmark ages can be found in Figures 3 and 4. As the landmark age increases, the most frequent optimal risk-assessment strategy shifts from every 10 years to every year for both genders. However, note that for women at landmark age 65 with high 5-year CVD risk at the landmark age the most frequent optimal risk-assessment schedule ranges from every 1 to 3 years. Similarly for men from landmark age 55 (Figure 4). There is a shift of the CVD risk between men and women. The numbers reported in Figures 3 and 4 are detailed in Section 3 of the online supplementary material.

### 4.2 Sensitivity analysis: exploring the effect of NB parameters

We perform a sensitivity analysis of the NB optimization with respect to the NB parameters *λ*, $u_{s}$, $c_{s}$, $c_{\nu }$. In general, we observe that results are robust with respect to the parameters choice and minor expected changes are observed. Specifically, *λ* increases, the 10-year frequency is optimal for fewer people, while intermediate frequency becomes optimal for a larger proportion of people. A similar observation can be done for the utility associated to statins $u_{s}$, the lower the impact of statins on the quality of life, the less preferred is the 10-year risk assessment. On the contrary, the higher the price of statins, $c_{s}$, the more risk-assessment strategies associated with less frequent visits are to be preferred.

The complete results of the sensitivity analysis are reported in Section 5 of the online supplementary material.

## 5 Discussion

In this paper, we introduced a novel statistical approach to address the multi-faceted problem of identifying optimal risk-assessment strategies for CVD risk prevention. Different CVD risk prevention strategies, such as habit/diet modification and statin prescription, and different risk-assessment schedules have been recommended worldwide (Lalor et al., 2012; NICE, 2014; Pylypchuk et al., 2018). In this work, we focussed on statin initiation because statins have been proven to be the most common CVD prevention method (Reiner, 2013) and we focussed on the UK NICE guidelines (NICE, 2014). The method that we are proposing refers to healthcare systems where a decision has to be made about balancing costs of frequent risk assessment against the benefits in terms of CVD prevention. However, those countries where risk assessments are already planned yearly and there is no issue in terms of resources, may not benefit from the findings of this work.

The novelty introduced in this work is two-fold: first, we provided an extension to the 2-stage landmarking model (Paige et al., 2018) in order to estimate the exact time at which the 5-year CVD risk exceeds the 5% threshold; second, we defined a net benefit function to discriminate among different visit schemes in order to assess the optimal CVD risk-assessment schedule per person at different landmark ages.

The extension of the 2-stage landmarking model consisted of defining a series of landmark sub-cohorts based on a set of prediction times of interest; of estimating BLUPs and of fitting a Cox model based on both fixed covariates and BLUPs, at each prediction time of interest. Future work could explore a similar extension to the landmarking 2.0 approach of Putter and van Houwelingen (2022).

The net benefit function is based on the difference between benefits (i.e. CVD-free life years) and costs (i.e. quality of life reduction, cost of the visits and of statins purchase) and it is designed as a landmark and person-specific function of the risk-assessment schedule $\tau^{f}$. The optimal CVD risk-assessment schedule for the *i*th person ($\tau_{i,L_{a}}^{opt}$) is the one associated with the highest NB value.

We applied the proposed model to an electronic health record dataset obtained through linking CPRD data to secondary care admissions from HES and mortality records from the ONS. According to our findings, only a portion of the cohort is expected to cross the 5% threshold and the proportion of this group of people increases with age. Since women have lower CVD incidence than men, more so at younger ages, then assessing CVD risk every 5 years, starting from age 40 for both men and women may be a sub-optimal strategy. Using our method we were able to recommend for each individual at each landmark age the optimal risk-assessment schedule. For lower risk categories with 5-year risk less than 3.75%, we found that assessing the CVD risk every 10 years is the most frequent optimal choice, while more frequent risk-assessment strategies of every 1 or 2 years were found to be optimal for the majority of the landmark cohort at higher risk. Note that almost all women older than 75 and men older than 70 are labelled as very high risk. This is in line with the fact that age is the most important risk factor for CVD diagnosis.

We had to make some assumptions in order to investigate this complex problem. These assumptions may be limitations of the present study, but also identify directions for further research. For example, we assumed that risk-assessment frequencies are maintained over the 10-year time horizon. We also assumed that each person starting statin therapy will be fully compliant, even though statin non-adherence is a well known issue (Simpson & Mendys, 2010). Another assumption of our model consisted of censoring deaths both for the identification of the time of crossing the threshold and for the NB computation. This choice is in accordance with the NICE guidelines (NICE, 2014). Fourth, we assume a linear trend for the longitudinal CVD risk factors, which may not be appropriate for predicting up to 10 years ahead. Finally, we defined a quite general NB function to identify an optimal risk-assessment schedule for a general population. However, a full economic assessment over the long-term (>10 years) could include broader economic and health implications, such as the cost to the health service of additional interventions (e.g. stents) and CVD events due to long periods of elevated CVD risk and time spent living with CVD. Also, the NB function is not able to deal with those people that are labelled as very high risk (5-year CVD risk at a specific landmark age greater than 5%), and separate recommendations are required for management of CVD risk in this population.

Future work will further explore these limitations. It is possible to adjust for statin non-adherence by providing a modified *θ*, or even a time dependent *θ*. The linear assumption behind the endogenous longitudinal variable can be improved by fitting more flexible mixed effects models, although this may require more complete and frequent measurements than are available in the CPRD dataset. To address the competing risk of death and account for time spent living with CVD, a competing risk or a multi-state model could be defined to assess CVD-specific risk. A more complex NB function could be designed to take into account both CVD and death. Our health outcome included only event-free life years up to 10 years adjusted for quality of life on statins, and we assumed that the cost per QALY gained used by NICE is applicable to these restricted outcomes. Another possible extension of the NB function could be designed for elder populations, that are completely labelled as very high risk. In this case, the risk-assessment strategy could recommend the *type of measurement* to be taken (i.e. blood tests, SBP, etc.), instead of the risk-assessment schedule.

## Supplementary Material

qnae086_Supplementary_Data

## Data Availability

The data were provided by the Clinical Practice Research Datalink (CPRD) under license from the UK Medicines and Healthcare products Regulatory Agency (protocol 162RMn2), and so are not publicly available. Access to CPRD data is subject to protocol approval via CPRD’s Research Data Governance (RDG) Process. Code is publicly available at https://github.com/fgaspe04/CPRD/.
