# Supplementary material for: Optimal risk-assessment scheduling for primary prevention of cardiovascular disease
Source: J R Stat Soc Ser A Stat Soc. 2024 Sep 17;188(3):920–34. doi: 10.1093/jrsssa/qnae086 (PMC12256122; doi:10.1093/jrsssa/qnae086)
Supplement: qnae086_Supplementary_Data [file qnae086_supplementary_data.pdf]

Supporting information for ”Optimal risk-assessment scheduling for primary prevention of Cardiovascular disease”

Francesca Gasperoni[1], Christopher H. Jackson[1], Angela M. Wood[2−8], Michael J. Sweeting[9], Paul Newcombe[1], David Stevens[10,11], and Jessica K. Barrett[1,*]

[1]MRC Biostatistics Unit, University of Cambridge, Cambridge, U.K.
[2]British Heart Foundation Cardiovascular Epidemiology Unit, Department of Public Health and Primary Care, University of Cambridge, Cambridge, U.K.
[3]Victor Phillip Dahdaleh Heart and Lung Research Institute, University of Cambridge, Cambridge, U.K.
[4]British Heart Foundation Centre of Research Excellence, University of Cambridge, Cambridge, U.K.
[5]National Institute for Health and Care Research Blood and Transplant Research Unit in Donor Health and Behaviour,University of Cambridge, Cambridge, U.K.
[6]Health Data Research UK Cambridge, Wellcome Genome Campus and University of Cambridge, Cambridge, U.K.
[7]Cambridge Centre of Artificial Intelligence in Medicine, University of Cambridge, Cambridge, U.K.
[8]British Heart Foundation Data Science Centre, Health Data Research UK, London, U.K.
[9]Department of Health Sciences, University of Leicester, Leicester, U.K.
[10]Liverpool Centre for Cardiovascular Science, University of Liverpool, Liverpool Heart & Chest Hospital, UK.
[11]Department of Cardiovascular and Metabolic Medicine, Institute of Life Course and Medical Sciences, University of Liverpool, UK.

August 10, 2024

1 Cohort selection, risk factors and outcome definitions

In this section we report details related to cohort selection and variables included in the proposed model.

In Figure 1, we represent the scheme of the cohort selection. The final derivation dataset is composed of 1,337,380 people distributed across 263 practices in the UK; while the validation dataset is composed of 633,622 people distributed in 135 practices.

Following Xu et al. [2021], we define the *study entry* for each person as the latest of the following four dates: the date of 6 months after registration at the general practice; the date the individual turned 30 years of age; the date that the data for the practice were up to standard [Tate et al., 2017]; or April 01, 2004, the date that the Quality and Outcomes Framework (QOF) was introduced [National Health Service, 2011]. We define the *study exit* for each person as the earliest of the following dates: the date of deregistration at the practice; the individual's death; the date that the individual turned 95 years of age; the last contact date for the practice with CPRD; or the administration end date (November 2017).

The Read codes (used to identify outcomes in CPRD) and International Classification of Diseases, Tenth Revision, codes (used to identify outcomes in primary or secondary diagnosis fields from Hospital Episode Statistics and in underlying or subordinate cause of death fields from the Office for National Statistics) are provided in the Web Appendix 1, Web Tables 1 and 2 of Xu et al. [2021].

Previous diagnosis of diabetes, renal disease, depression, migraine, severe mental illness, rheumatoid arthritis and atrial fibrillation are ascertained from CPRD Read codes. Blood pressure medication (yes/no) is ascertained from CPRD prescription information and it is defined as the date of first prescription. Statin initiation is defined

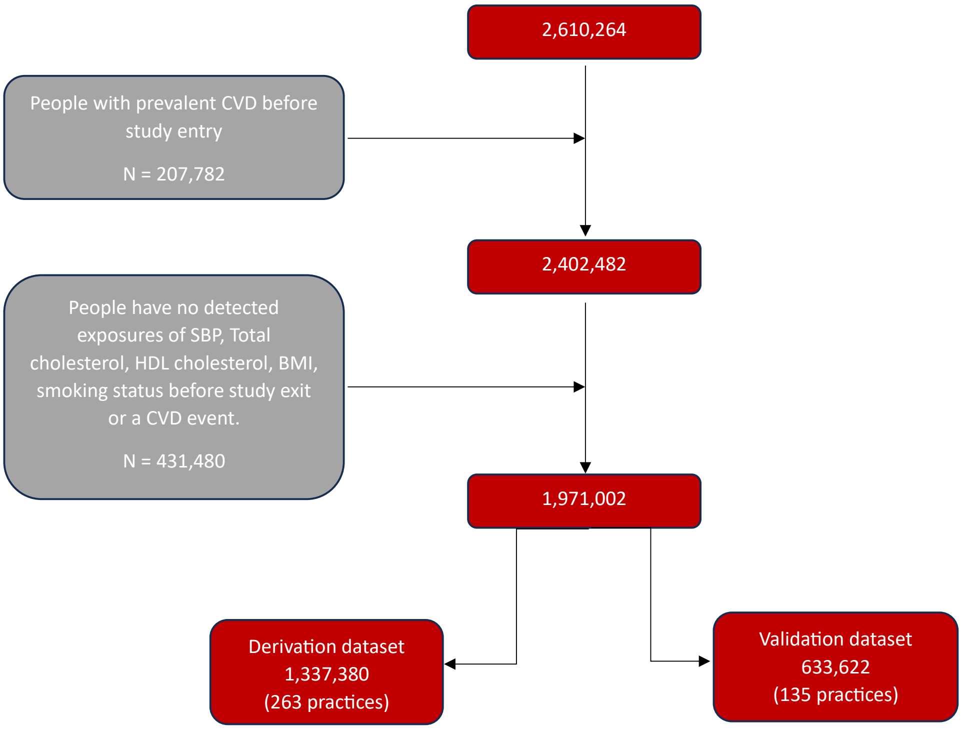

Web Figure 1: Flow chart of the selection process of the analysed cohort.

as the date of first CPRD prescription (code list for CPRD prescription provided in Web Appendix 2, Web Table 3 of Xu et al. [2021]). Finally, Townsend deprivation index ranges from 1 to 20. This index presents a total of 1979 missing values (0.08% of the whole cohort), that are imputed through the mean value per each landmark.

We filter out biologically implausible values: BMI > 80 kg/m^2 ; SBP > 250 mmHg or < 60 mmHg; total cholesterol level > 20 mmol/L or < 1.75 mmol/L; HDL cholesterol level > 3.1 mmol/L or < 0.3 mmol/L.

Furthermore, we consider two different sets of risk factors, if we are performing the analysis before or after landmark age 60. At all landmark ages, we include the BLUPs of BMI, HDL, SBP, total cholesterol and smoking. At all landmark ages, we include blood pressure medication, Townsend deprivation index, previous diagnosis of diabetes, depression, migraine and severe mental illness. Previous diagnosis of renal disease, rheumatoid arthritis and atrial fibrillation are included only after landmark age 60. This choice is motivated by the fact that these specific conditions are extremely rare at younger ages and the estimates of Cox model in Eq. 12 of the main manuscript are unfeasible.

2 Details of the Incremental Net Benefit function

2.1 Derivation of EFLY before and after statins initiation

We evaluate as benefit the restricted event free life years (EFLY), i.e. we investigate the time to CVD diagnosis, T, restricted to the observable time window $[L_a, L_a + 10]$.

To define the restricted EFLY, $min(T, L_a + 10)$, we assume that the time to CVD can be written as $T = T^{NS} + T^S$, where T^{NS} is event-free time elapsed before statin initiation and T^S is the event-free time elapsed after statin initiation. The definition of the distribution of the time to CVD, T, should reflect the fact that statin usage has a positive impact on CVD-free life expectancy [Ferket et al., 2012]. In order to express this gain in EFLY, we quantify the effect of statins via the hazard ratio θ. In this article, we set $\theta = 0.8$, following previous meta-analysis of statin trials [Unit Epidemiological Studies, 2005].

The discontinuity point in the definition of T, that separates the time to CVD without statins T^{NS} and the

time with statins T^S, is defined as $\tau_{k^*_{i,L_a}}$, the first visit among the scheduled ones $\boldsymbol{\tau}$ that happens after t^*_{i,L_a}, the predicted time when the 5-year CVD risk of person i exceeds the 5% threshold at landmark age L_a. $\tau_{k^*_{i,L_a}}$ is landmark and person-specific and depends on the risk-assessment strategy under evaluation, $\boldsymbol{\tau}$.

We can define the hazard rate of CVD onset, given the personal covariates known at time L_a and $\tau_{k^*_i}$ as reported in Eq. (1).

$$\begin{aligned}\lambda(t;\mathbf{x}_i(L_a),L_a,\tau_{k^*_{i,L_a}}) &= \lambda^{NS}(t;\mathbf{x}_i(L_a),L_a)\cdot\mathbb{1}\{t\le\tau_{k^*_{i,L_a}}\}+\lambda^{S}(t;\mathbf{x}_i(L_a),L_a)\cdot\mathbb{1}\{t>\tau_{k^*_{i,L_a}}\}\\ &= \lambda_0(t;L_a)\cdot\exp\{\mathbf{x}_i(L_a)^T\boldsymbol{\beta}(L_a)\}\cdot\Big[\mathbb{1}\{t\le\tau_{k^*_{i,L_a}}\}+\theta\cdot\mathbb{1}\{t>\tau_{k^*_{i,L_a}}\}\Big],\quad t\ge L_a.\end{aligned}\tag{1}$$

We can compute the cumulative hazard function $\Lambda(t;\mathbf{x}_i(L_a),L_a,\tau_{k^*_{i,L_a}})$ as explained in Eq. (2).

$$\begin{aligned}\Lambda(t;\mathbf{x}_i(L_a),L_a,\tau_{k^*_{i,L_a}}) &= \int_{L_a}^{t}\lambda_0(u)\cdot\exp\{\mathbf{x}_i(L_a)^T\boldsymbol{\beta}(L_a)\}\cdot\Big[\mathbb{1}\{u\le\tau_{k^*_{i,L_a}}\}+\theta\cdot\mathbb{1}\{u>\tau_{k^*_{i,L_a}}\}\Big]\,du\\ &= \begin{cases}\Lambda_0(t;L_a)\exp\{\mathbf{x}_i(L_a)^T\boldsymbol{\beta}(L_a)\}, & t\le\tau_{k^*_{i,L_a}}\\ \Lambda_0(\tau_{k^*_{i,L_a}})\exp\{\mathbf{x}_i(L_a)^T\boldsymbol{\beta}(L_a)\}+(\Lambda_0(t;L_a)-\Lambda_0(\tau_{k^*_{i,L_a}}))\cdot\theta\cdot\exp\{\mathbf{x}_i(L_a)^T\boldsymbol{\beta}(L_a)\}, & t>\tau_{k^*_{i,L_a}}\end{cases}\end{aligned}\tag{2}$$

Defining $\Lambda^{NS}(t;\mathbf{x}_i(L_a),L_a)$ as $\Lambda_0(t;L_a)\exp\{\mathbf{x}_i(L_a)^T\boldsymbol{\beta}(L_a)\}$, we can rewrite Eq. (2) as follows:

$$\Lambda(t;\mathbf{x}_i(L_a),L_a,\tau_{k^*_{i,L_a}}) = \begin{cases}\Lambda^{NS}(t;\mathbf{x}_i(L_a),L_a), & t\le\tau_{k^*_{i,L_a}}\\ \Lambda^{NS}(\tau_{k^*_{i,L_a}};\mathbf{x}_i(L_a),L_a)+(\Lambda^{NS}(t;\mathbf{x}_i(L_a),L_a)-\Lambda^{NS}(\tau_{k^*_{i,L_a}};\mathbf{x}_i(L_a),L_a))\cdot\theta, & t>\tau_{k^*_{i,L_a}}\end{cases}\tag{3}$$

Finally, we the survival function $S(t;\mathbf{x}_i(L_a),\tau_{k^*_{i,L_a}})$ in Eq. (4).

$$S(t;\mathbf{x}_i(L_a),L_a,\tau_{k^*_{i,L_a}}) = \exp\{-\Lambda(t;\mathbf{x}_i(L_a),\tau_{k^*_{i,L_a}},L_a)\} = \begin{cases}S^{NS}(t;\mathbf{x}_i(L_a),L_a), & t\le\tau_{k^*_{i,L_a}}\\ S^{S}(t;\mathbf{x}_i(L_a),L_a), & t>\tau_{k^*_{i,L_a}}\end{cases}\tag{4}$$

where:

$$S^S(t;\mathbf{x}_i(L_a),L_a) = S^{NS}(\tau_{k^*_{i,L_a}};\mathbf{x}_i(L_a),L_a)\cdot\left(\frac{S^{NS}(t;\mathbf{x}_i(L_a),L_a)}{S^{NS}(\tau_{k^*_{i,L_a}};\mathbf{x}_i(L_a),L_a)}\right)^{\theta}\tag{5}$$

Note that if a person is never prescribed statins, then we have $S(t;\mathbf{x}_i(L_a),L_a)=S^{NS}(t;\mathbf{x}_i(L_a),L_a)$. Conditioning on $\tau_{k^*_{i,L_a}}$, $\tau_{k^*_{i,L_a}}\le L_a+10$, we are able to define the restricted EFLY in Eq. (6).

$$\begin{aligned}EFLY &= EFLY_{NS}(\tau_{k^*_{i,L_a}})+EFLY_S(\tau_{k^*_{i,L_a}})\\ &= \int_{L_a}^{\tau_{k^*_{i,L_a}}}S^{NS}(t;\mathbf{x}_i(L_a),L_a)\,dt+\int_{\tau_{k^*_{i,L_a}}}^{L_a+10}S^{S}(t;\mathbf{x}_i(L_a),L_a)\,dt.\end{aligned}\tag{6}$$

3 Optimal risk-assessment scheduling: CVD free life years

In this section, we report the recommended risk-assessment strategies across different landmark ages for women in Table 1 and for men in Table 2. In each table, we describe the results based on the landmark ages (values in columns) and on the 5-year CVD risk categories estimated at each landmark age (values in rows). The percentage associated to each number of the table is computed with respect to the landmark cohort. For example, if we focus on women at high risk at landmark age 40 (third column from the left in Table 1), we note that 78 (0.05% of 155497) women are recommended to have a risk-assessment every year. Furthermore, we report the total number of people belonging to each risk category and the total number of people among them, whose 5-year CVD risk is not expected to cross the 5% threshold in the next 10 years. These two numbers are at the bottom of each risk category block. For example, if we focus on women at high risk at landmark age 40 (third column from the left in Table 1), we note that 483 are labelled as High risk and 56 (11.59%) of them are not expected to cross the 5% threshold. At the bottom of both tables we record the landmark cohort size and total number of people whose 5-year CVD risk is not expected to cross the 5% threshold in the next 10 years. Looking at these rows, we note that the landmark cohort size decreases over landmark ages. The total number of people whose 5% CVD risk is not expected to cross the threshold decreases over landmark ages. This line should be read in pair with the top one where the total number of people labelled as very high risk is reported (note that these numbers increase over landmark ages). These observations hold for both women and men.

Relevant differences between women in Table 1 and men in Table 2 are related to the time when the number of people whose 5-year CVD risk is not expected to cross the threshold starts dropping (last row) and the time when the percentage of people labelled as very high risk starts increasing (top row). Indeed, for women the first number start dropping at landmark age 65 (from 34.82% to 8.09%), while for men at landmark age 55 (from 43.02% to 5.38%). Analogously, almost a quarter of the age 65 landmark cohort of women (24.61%) is labelled as very high risk, similar number is reached by men already at landmark age 55 (28.06%).

Web Table 1:

Optimal CVD-risk assessment frequency across landmark ages (in columns), stratified by baseline risk category (in rows). This table refers to women and the outcome of interest is 10-year CVD. In the two bottom lines we report the landmark cohort size and the total number of people whose 5-year CVD risk is not expected to cross the 5% threshold in the next 10 years.

Risk class	Optimal	40	45	50	55	60	65	70	75	80
Very high	-	360 (0.23%)	1110 (0.69%)	3580 (2.47%)	5065 (4.11%)	11953 (10.95%)	21263 (24.61%)	46914 (74.16%)	46362 (99.09%)	37178 (100%)
High	1	78 (0.05%)	411 (0.26%)	865 (0.6%)	1487 (1.21%)	1999 (1.83%)	5771 (6.68%)	7882 (12.46%)	306 (0.65%)	-
	2	85 (0.05%)	474 (0.3%)	968 (0.67%)	1749 (1.42%)	1969 (1.8%)	7015 (8.12%)	4832 (7.64%)	94 (0.2%)	-
	3	63 (0.04%)	97 (0.06%)	631 (0.44%)	1002 (0.81%)	1989 (1.82%)	4438 (5.14%)	1430 (2.26%)	11 (0.02%)	-
	4	6 (0%)	143 (0.09%)	157 (0.11%)	881 (0.72%)	1768 (1.62%)	452 (0.52%)	947 (1.5%)	-	-
	5	79 (0.05%)	69 (0.04%)	399 (0.28%)	224 (0.18%)	2091 (1.92%)	367 (0.42%)	3 (0%)	2 (0%)	-
	6	63 (0.04%)	142 (0.09%)	253 (0.17%)	502 (0.41%)	2028 (1.86%)	11 (0.01%)	-	-	-
	7	-	3 (0%)	23 (0.02%)	109 (0.09%)	-	-	-	-	-
	8	-	-	-	-	-	-	-	-	-
	9	-	-	-	-	-	-	-	-	-
	10	109 (0.07%)	80 (0.05%)	983 (0.68%)	509 (0.41%)	1498 (1.37%)	603 (0.7%)	114 (0.18%)	-	-
	Total	483	1419	4279	6463	13342	18657	15208	413	0
	Never cross	56 (11.59%)	4 (0.28%)	432 (10.1%)	89 (1.38%)	4 (0.03%)	109 (0.58%)	2 (0.01%)	-	-
Med-high	1	7 (0%)	5 (0%)	1 (0%)	12 (0.01%)	1 (0%)	-	-	-	-
	2	49 (0.03%)	228 (0.14%)	101 (0.07%)	128 (0.1%)	57 (0.05%)	1428 (1.65%)	110 (0.17%)	1 (0%)	-
	3	59 (0.04%)	437 (0.27%)	173 (0.12%)	932 (0.76%)	348 (0.32%)	4890 (5.66%)	150 (0.24%)	6 (0.01%)	-
	4	32 (0.02%)	750 (0.47%)	535 (0.37%)	1820 (1.48%)	780 (0.71%)	5926 (6.86%)	462 (0.73%)	3 (0.01%)	-
	5	98 (0.06%)	560 (0.35%)	1172 (0.81%)	1617 (1.31%)	1881 (1.72%)	7889 (9.13%)	-	-	-
	6	10 (0.01%)	394 (0.25%)	479 (0.33%)	823 (0.67%)	6 (0.01%)	-	-	-	-
	7	-	-	-	2 (0%)	-	-	-	-	-
	8	-	-	-	-	-	-	-	-	-
	9	-	-	-	-	-	-	-	-	-
	10	1323 (0.85%)	1870 (1.17%)	8943 (6.17%)	12398 (10.07%)	27151 (24.87%)	11221 (12.99%)	413 (0.65%)	2 (0%)	-
	Total	1578	4244	11404	17732	30224	31354	1135	12	0
	Never cross	844 (53.49%)	588 (13.85%)	4887 (42.85%)	7727 (43.58%)	1113 (3.68%)	1417 (4.52%)	1 (0.09%)	-	-
Med-low	1	-	-	-	-	-	-	-	-	-
	2	5 (0%)	-	-	-	-	-	-	-	-
	3	11 (0.01%)	51 (0.03%)	1 (0%)	36 (0.03%)	-	14 (0.02%)	-	-	-
	4	32 (0.02%)	247 (0.15%)	13 (0.01%)	167 (0.14%)	13 (0.01%)	146 (0.17%)	-	-	-
	5	-	185 (0.12%)	8 (0.01%)	127 (0.1%)	-	-	-	-	-
	6	-	-	-	-	-	-	-	-	-
	7	-	-	-	-	-	-	-	-	-
	8	-	-	-	-	-	-	-	-	-
	9	-	-	-	-	-	-	-	-	-
	10	8609 (5.54%)	20096 (12.56%)	40250 (27.78%)	56967 (46.26%)	50054 (45.85%)	14949 (17.3%)	-	-	-
	Total	8657	20579	40272	57297	50067	15109	0	0	0
	Never cross	7702 (88.97%)	16316 (79.28%)	32131 (79.78%)	54380 (94.91%)	33429 (66.77%)	5442 (36.02%)	-	-	-
Low	1	-	-	-	-	-	-	-	-	-
	2	-	-	-	-	-	-	-	-	-
	3	-	-	-	-	-	-	-	-	-
	4	-	-	-	-	-	-	-	-	-
	5	-	-	-	-	-	-	-	-	-
	6	-	-	-	-	-	-	-	-	-
	7	-	-	-	-	-	-	-	-	-
	8	-	-	-	-	-	-	-	-	-
	9	-	-	-	-	-	-	-	-	-
	10	144419 (92.88%)	132623 (82.9%)	85372 (58.92%)	36578 (29.71%)	3587 (3.29%)	20 (0.02%)	-	-	-
	Total	144419	132623	85372	36578	3587	20	0	0	0
	Never cross	143891 (99.63%)	131784 (99.37%)	81151 (95.06%)	36558 (99.95%)	3468 (96.68%)	20 (100%)	-	-	-
	Total	155497	159975	144907	123135	109173	86403	63257	46787	37178
	Never cross	152493 (98.07%)	148692 (92.95%)	118601 (81.85%)	98754 (80.2%)	38014 (34.82%)	6988 (8.09%)	3 (0%)	-	-

In Figure 2 we report a detailed representation of risk profiles, $\hat{r}_i(s+5; \mathbf{x}_i(s), s)$ $s \in \{40, \ldots, 50\}$, for women at $L_a = 40$, whose 5-year CVD risk at $L_a = 40$ is classified as high. It is immediate to notice that the median $\tau_{k^*_{i,40}}$ (black solid lines) increases according to the optimal frequency recommendation. Indeed, people whose 5-year CVD risk is expected to exceed the 5% threshold later in time are more likely to be recommended a lower frequency risk-assessment strategy. Furthermore, it is interesting to notice that the 5-year CVD risk estimated at the baseline is relevant for the risk-assessment recommendation, but the information given by the risk profile is fundamental for the risk-assessment recommendation. Indeed, we observe that different risk-profile trends (more steep or more flat) can lead to opposite risk-assessment recommendation even for people at high risk of CVD.

3.1 Descriptive statistics of the landmark cohorts

In this Section, we report the number of CVD diagnoses and statins intake across gender and landmark ages (see Table 3). For example, if we focus on women at landmark age 40, we observe that 1022 will have a CVD diagnoses and 3922 will start statins intake after age 40. We also report the descriptive characteristics of each

Web Table 2:

Optimal CVD-risk assessment frequency across landmark ages (in columns), stratified by baseline risk category (in rows). This table refers to men and the outcome of interest is 10-year CVD. In the two bottom lines we report the landmark cohort size and the total number of people whose 5-year CVD risk is not expected to cross the 5% threshold in the next 10 years.

Risk class	Optimal	40	45	50	55	60	65	70	75	80
Very high	-	767 (0.58%)	4053 (2.87%)	13792 (10.73%)	29967 (28.06%)	56201 (60.96%)	63064 (93.03%)	45487 (99.95%)	30978 (100%)	23189 (100%)
High	1	171 (0.13%)	1395 (0.99%)	3898 (3.03%)	7673 (7.19%)	10328 (11.2%)	2904 (4.28%)	22 (0.05%)	-	-
	2	291 (0.22%)	1376 (0.97%)	4921 (3.83%)	7407 (6.94%)	7386 (8.01%)	1335 (1.97%)	3 (0.01%)	-	-
	3	251 (0.19%)	636 (0.45%)	4853 (3.78%)	10006 (9.37%)	3384 (3.67%)	24 (0.04%)	-	-	-
	4	149 (0.11%)	331 (0.23%)	677 (0.53%)	4137 (3.87%)	5603 (6.08%)	27 (0.04%)	-	-	-
	5	79 (0.06%)	393 (0.28%)	168 (0.13%)	924 (0.87%)	255 (0.28%)	-	-	-	-
	6	31 (0.02%)	81 (0.06%)	212 (0.16%)	9 (0.01%)	-	-	-	-	-
	7	15 (0.01%)	108 (0.08%)	82 (0.06%)	-	-	-	-	-	-
	8	-	-	-	-	-	-	-	-	-
	9	-	-	-	-	-	-	-	-	-
	10	8 (0.01%)	212 (0.15%)	97 (0.08%)	96 (0.09%)	897 (0.97%)	2 (0%)	-	-	-
	Total	995	4532	14908	30252	27853	4292	25	0	0
	Never cross	3 (0.3%)	132 (2.91%)	70 (0.47%)	9 (0.03%)	59 (0.21%)	-	-	-	-
Med-high	1	2 (0%)	22 (0.02%)	-	1 (0%)	-	-	-	-	-
	2	91 (0.07%)	214 (0.15%)	823 (0.64%)	65 (0.06%)	9 (0.01%)	92 (0.14%)	-	-	-
	3	194 (0.15%)	1210 (0.86%)	4037 (3.14%)	380 (0.36%)	75 (0.08%)	71 (0.1%)	-	-	-
	4	349 (0.27%)	2788 (1.97%)	7091 (5.52%)	6738 (6.31%)	941 (1.02%)	145 (0.21%)	-	-	-
	5	786 (0.6%)	3918 (2.77%)	10381 (8.08%)	9182 (8.6%)	-	-	-	-	-
	6	685 (0.52%)	976 (0.69%)	7200 (5.6%)	-	-	-	-	-	-
	7	233 (0.18%)	154 (0.11%)	44 (0.03%)	-	-	-	-	-	-
	8	-	-	-	-	-	-	-	-	-
	9	-	-	-	-	-	-	-	-	-
	10	730 (0.55%)	5018 (3.55%)	8599 (6.69%)	22467 (21.04%)	6531 (7.08%)	124 (0.18%)	-	-	-
	Total	3070	14300	38175	38833	7556	432	0	0	0
	Never cross	112 (3.65%)	1845 (12.9%)	2236 (5.86%)	2077 (5.35%)	959 (12.69%)	-	-	-	-
Med-low	1	-	-	-	-	-	-	-	-	-
	2	4 (0%)	-	-	-	-	-	-	-	-
	3	11 (0.01%)	-	11 (0.01%)	1 (0%)	-	-	-	-	-
	4	259 (0.2%)	129 (0.09%)	237 (0.18%)	6 (0.01%)	-	-	-	-	-
	5	544 (0.41%)	2175 (1.54%)	494 (0.38%)	1 (0%)	-	-	-	-	-
	6	849 (0.65%)	254 (0.18%)	47 (0.04%)	-	-	-	-	-	-
	7	-	-	-	-	-	-	-	-	-
	8	-	-	-	-	-	-	-	-	-
	9	-	-	-	-	-	-	-	-	-
	10	22154 (16.84%)	71320 (50.47%)	55334 (43.05%)	7579 (7.1%)	587 (0.64%)	2 (0%)	-	-	-
	Total	23821	73878	56123	7587	587	2	0	0	0
	Never cross	15171 (63.69%)	59846 (81.01%)	47455 (84.56%)	3539 (46.65%)	511 (87.05%)	-	-	-	-
Low	1	-	-	-	-	-	-	-	-	-
	2	-	-	-	-	-	-	-	-	-
	3	-	-	-	-	-	-	-	-	-
	4	-	-	-	-	-	-	-	-	-
	5	-	-	-	-	-	-	-	-	-
	6	-	-	-	-	-	-	-	-	-
	7	-	-	-	-	-	-	-	-	-
	8	-	-	-	-	-	-	-	-	-
	9	-	-	-	-	-	-	-	-	-
	10	102895 (78.22%)	44541 (31.52%)	5549 (4.32%)	149 (0.14%)	3 (0%)	-	-	-	-
	Total	102895	44541	5549	149	3	0	0	0	0
	Never cross	100271 (97.45%)	44125 (99.07%)	5541 (99.86%)	115 (77.18%)	3 (100%)	-	-	-	-
	Total	131548	141304	128547	106788	92200	67790	45512	30978	23189
	Never cross	115557 (87.84%)	105948 (74.98%)	55302 (43.02%)	5740 (5.38%)	1532 (1.66%)	-	-	-	-

landmark cohort, stratified by the 5-year CVD risk classification at the landmark age. Descriptive statistics are reported in Table 4 and 5 for women and men respectively. We observe a higher risk for people under blood pressure medication, with diagnoses of depression, diabetes, migraine, renal disease, rheumatoid arthritis, severe mental illness and systemic lupus eritematosus. Higher values of SBP, total cholesterol (TCHOL), BMI are associated with people at higher risk. The Townsend 20 index and smoking are also associated with higher risk.

Web Table 3:

Cardiovascular disease diagnosis and statins intake across landmark ages and gender.

	Gender	40	45	50	55	60	65	70	75	80
CVD	Women	1022 (0.66 %)	1701 (1.06 %)	2286 (1.58 %)	2836 (2.3 %)	3760 (3.44 %)	4081 (4.72 %)	4688 (7.41 %)	5175 (11.06 %)	5410 (14.55 %)
	Men	1799 (1.37 %)	3074 (2.18 %)	4425 (3.44 %)	5338 (5 %)	6244 (6.77 %)	5663 (8.35 %)	5297 (11.64 %)	4860 (15.69 %)	4266 (18.4 %)
Statins	Women	3922 (2.52 %)	7056 (4.41 %)	10241 (7.07 %)	13253 (10.76 %)	16819 (15.41 %)	16091 (18.62 %)	13860 (21.91 %)	9470 (20.24 %)	5876 (15.81 %)
	Men	6981 (5.31 %)	11376 (8.05 %)	15135 (11.77 %)	17785 (16.65 %)	21480 (23.3 %)	17719 (26.14 %)	12062 (26.5 %)	6456 (20.84 %)	3749 (16.17 %)

4 Validation: C-index and Brier score

4.1 Validation

We validate the 2-stage landmark model for estimating the probability of not being diagnosed with CVD before statins initiation, described in Section 3.2 of the main manuscript. The estimated c-indices are represented via

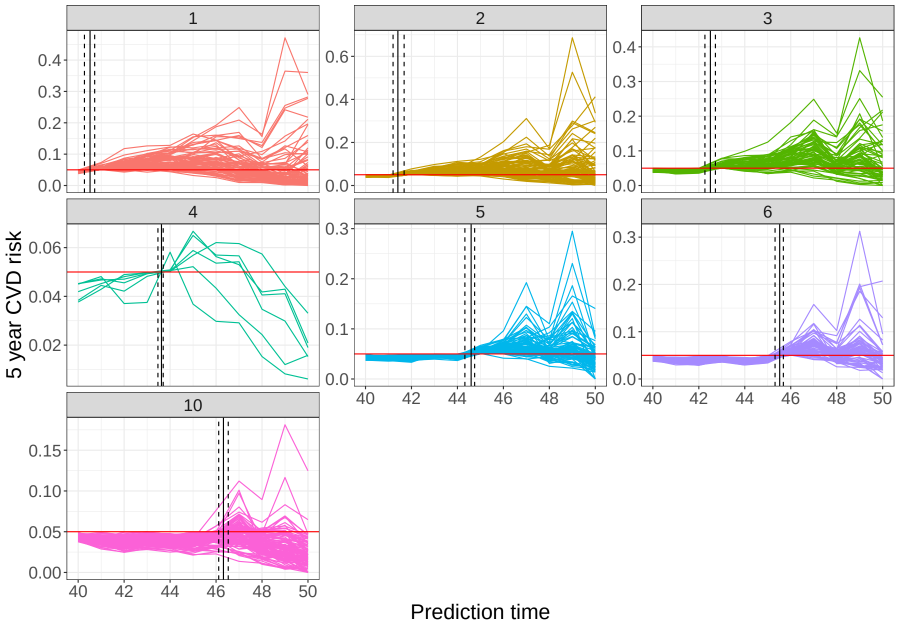

Web Figure 2: Risk profile representation for women at $L_a = 40$, whose 5-year CVD risk at $L_a = 40$ is classified as high. Each panel represents an optimal risk-assessment frequency. Each line is the estimated risk profile for a specific person. The solid black line represents the median expected time of crossing the 5% threshold (represented via the horizontal red line). The dashed lines represent the first and third quartiles of the $t_{k_i^*,40}$ distribution.

dots in Figure 3, while the Brier scores are represented in Figure 4.

In Figure 3, we found good overall discrimination (overall c-index equal to 0.78, represented via a solid black line).

Secondly, we validate the extended 2-stage landmarking approach described in Section 3.3 of the main manuscript and we report the estimated c-indices in Figure 5 and the estimated Brier Scores in Figure 6.

Note that the extended 2-stage landmarking approach for estimating t^*_{i,L_a} in general has good discriminatory power and good prediction accuracy (the lower the Brier Score, the higher the predictive accuracy of the model). The model performs better for women than men (at each time s the c-index for women is higher than the c-index for men, and the inverse for the Brier score). Furthermore, the model performance tends to decrease for higher landmark ages. Low c-indices with high standard deviations are found for older landmark ages (75, 80), for later prediction times $s \in \{83, 84, 85\}$ and $s \in \{88, 89, 90\}$ respectively. This could be due to the fact that the mean follow-up time is lower at higher landmark ages and very few people are observed after 83 years at landmark age 75 and after 88 years at landmark age 80.

We observe similar trends between Figure 3, Figure 4 and Figure 5, Figure 6: lower discrimination and prediction accuracy for the men landmark cohorts and a decline in model performance as the landmark age increases.

5 Sensitivity analysis: exploring the effect of NB parameters

We consider three sensitivity analyses by varying key parameters as follows:

- $\lambda \in [20,000; 30,000]$ £/year, while $u_s = 0.997$, $c_s = 150$ £/year, and $c_\nu = 18.39$ £/visit. The results are reported in panel A of Figure 7.
- $u_s \in [0.997; 1]$, while $\lambda = 25,000$ £/year, $c_s = 150$ £/year, and $c_\nu = 18.39$ £/visit. The results are reported in panel B of Figure 7.
- $c_s \in [4; 320]$ £/year, while $\lambda = 25,000$ £/year, $u_s = 0.997$, and $c_\nu = 18.39$ £/visit. The results are reported in panel C of Figure 7.

In general, we observe that results are robust with respect to the parameter choice.

In panel A of Figure 7, we vary the value of λ from 20,000 £/year to 30,000 £/year, as λ increases, the 10-year frequency is optimal for fewer people, while intermediate frequency (such as 4-7 years) becomes optimal for a larger proportion of people. Visits every 1, 2, 3 years are optimal for a constant proportion of the cohort.

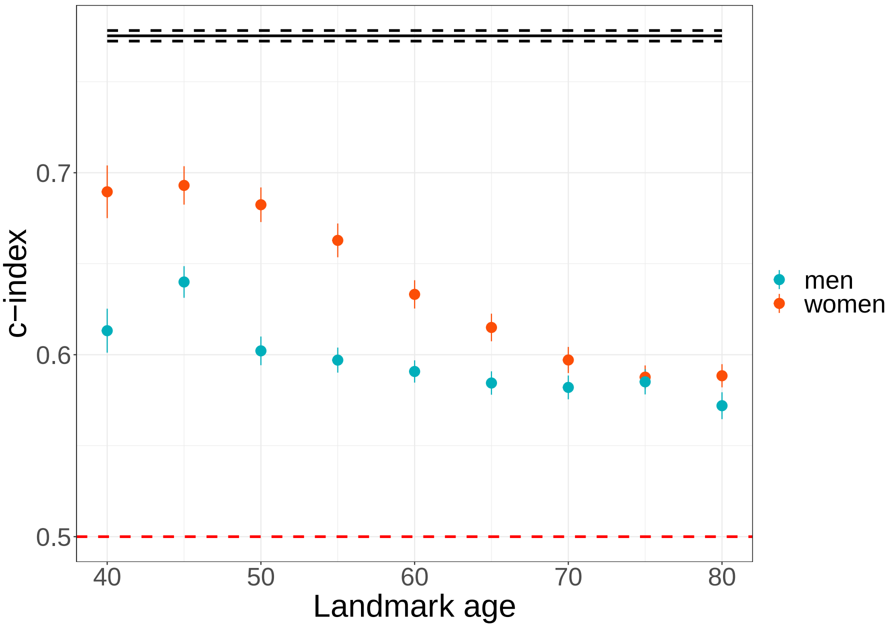

Web Figure 3: Estimated c-indices for the second landmark model for men (blue dots) and women (red dots) for different values of starting time t. Each point represents a c-index computed for a specific $s = L_a \in \{40, 45, .., 80\}$ and $w = 10$, since we are interested in the discrimination accuracy of the 10-year CVD risk. Points at $L_a = 40$, represents the c-indices estimated with $s = 40$ and $w = 10$. The solid black lines represent the overall c-index across landmark ages and gender (dashed lines represent 95% confidence interval). The dashed red line at 0.5 represents the minimum sensible value of the c-index.

An increasing λ can be interpreted as a stronger willingness to pay for increased expectancy of CVD-free life years, so intermediate frequencies tend to be preferred over 10-yearly risk-assessments.

We see an analogous behaviour for u_s (panel B of Figure 7). We vary the utility factor from 0.997 (which implies a decrease of quality of life equal to 0.003) to 1 (which implies that taking statins has no effect at all on the quality of life). We notice that 1 to 4-year risk-assessment strategies are the optima for a constant number of people. We note when the impact of statins on quality of life is low (u_s tends to 1), the 10-year frequency schedule is less preferred, while intermediate frequencies (4-8 years) are preferred. If no burden is associated with taking statins ($u_s = 1$), then the optimal strategy is to initiate statins immediately.

In contrast, the higher the price of statins, c_s, the more risk-assessment strategies associated with less frequent visits are to be preferred (panel C of Figure 7). This is expected because higher costs imply decreased net benefit of statin usage.

We investigated also c_ν varying between 15 £/visit to 1000 £/visit (results not shown). Despite the broad range explored, the optimal schedule proportions are unchanging across all values of c_ν. This result is expected because this term of the NB is not comparable in scale with the terms associated with expected event free life years in Eq. (2) of the manuscript.

It is also immediate to notice from the range reported in the y-axis of Figure 7 that the greatest part of the whole cohort (>70%) is recommended to be assessed every 10 years. This is due to the fact that we are considering the stacked landmark cohorts and the biggest landmark cohorts are those ones collected at $L_a = 40$, $L_a = 45$, that are composed of younger and healthier people.

References

Bart S Ferket, Bob JH van Kempen, Jan Heeringa, Sandra Spronk, Kirsten E Fleischmann, Rogier LG Nijhuis, Albert Hofman, Ewout W Steyerberg, and MG Myriam Hunink. Personalized prediction of lifetime benefits with statin therapy for asymptomatic individuals: a modeling study. *PLoS Med*, 9(12):e1001361, 2012.

National Health Service. NHS digital QOF. `https://digital.nhs.uk/data-and-information/publications/statistical/quality-and-outcomes-framework-achievement-prevalence-and-exceptions-data/quality-and-outcomes-framework-2010-11`, 2011. Accessed: 2022-06-20.

A Rosemary Tate, Sheena Dungey, Simon Glew, Natalia Beloff, Rachael Williams, and Tim Williams. Quality of recording of diabetes in the uk: how does the gp's method of coding clinical data affect incidence estimates? cross-sectional study using the cprd database. *BMJ open*, 7(1), 2017.

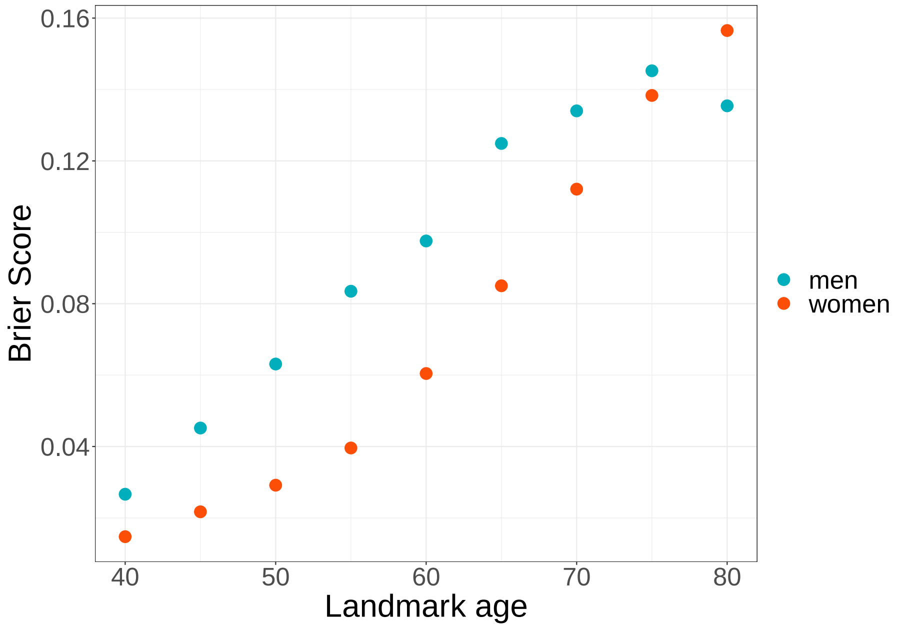

Web Figure 4: Estimates of Brier Score, $BS_s(w)$ where $s = L_a \in \{40, 45, .., 80\}$ and $w = 10$. Each $BS_{L_a}(10)$ is represented through a colored dot (blue dots for men and red dots for women).

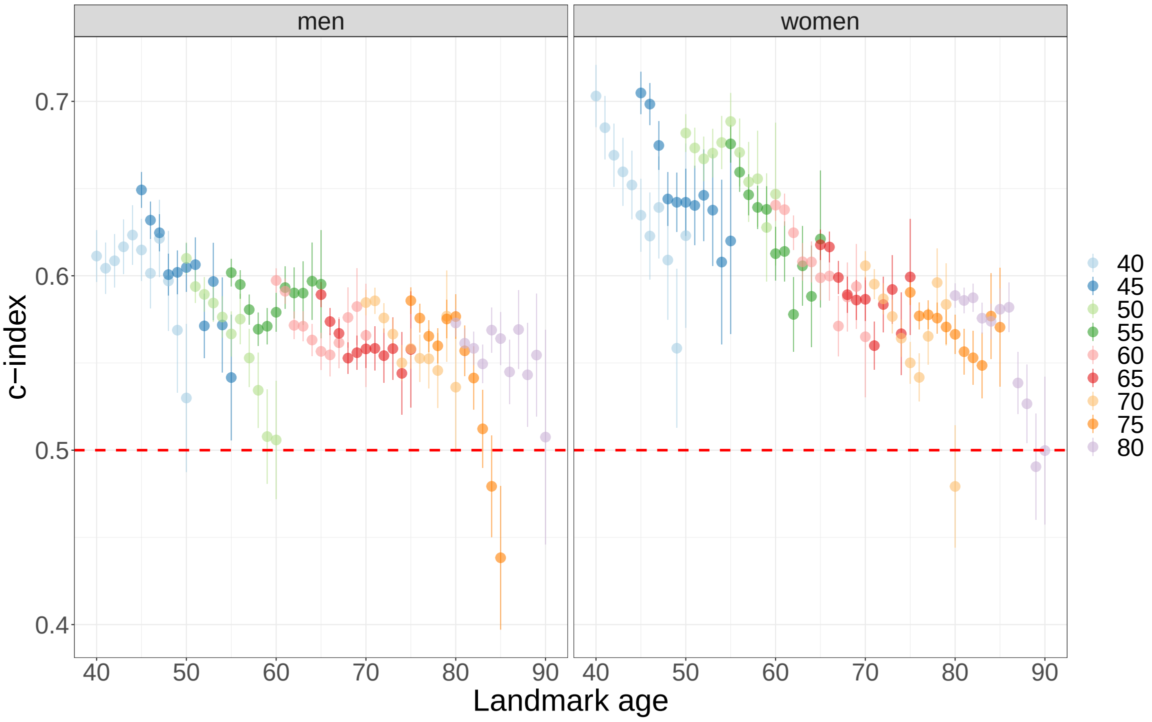

Web Figure 5: Estimated c-indices for the first landmark model for women (left panel) and men (right panels) for different values of starting time s. Each point represents a c-index computed for a specific $s \in \mathcal{P}_{L_a}$ and $w = 5$, since we are interested in the discrimination accuracy of the 5-year CVD risk. We associate a specific color to each landmark set. All points in light blue are associated with $L_a = 40$, and from the first point from the left we have $s \in \{40, 41, 42, .., 50\}$.The dashed red line at 0.5 represents the minimum sensible value of the c-index. Values lower than 0.5 are recorded at older ages, for the latest time-windows (i.e., 83-88, 84-89, 85-90 in orange for men, 88-93, 89-94, 90-95 in violet for men).

Unit Epidemiological Studies. Efficacy and safety of cholesterol-lowering treatment: prospective meta-analysis of data from 90 056 participants in 14 randomised trials of statins. *Lancet*, 366(9493):1267–1278, 2005.

Zhe Xu, Matthew Arnold, David Stevens, Stephen Kaptoge, Lisa Pennells, Michael J Sweeting, Jessica Barrett, Emanuele Di Angelantonio, and Angela M Wood. Prediction of cardiovascular disease risk accounting for

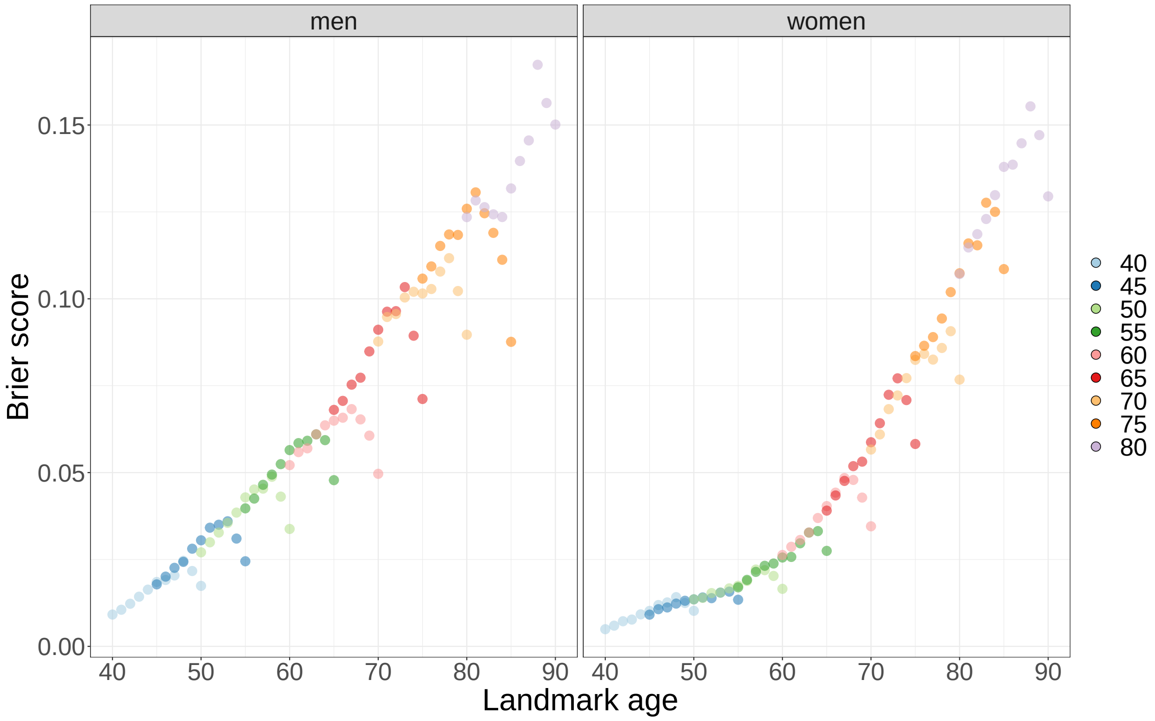

Web Figure 6: Estimates of Brier Score, $BS_s(w)$ where $s \in \mathcal{P}_{L_a}$ and $w = 5$. Each $BS_s(5)$ is represented through a colored dot (each landmark age is associated to a specific color). BS associated to women are reported in the left panel, while the BS associated to men are reported in the right panel.

future initiation of statin treatment. *American Journal of Epidemiology*, 2021.

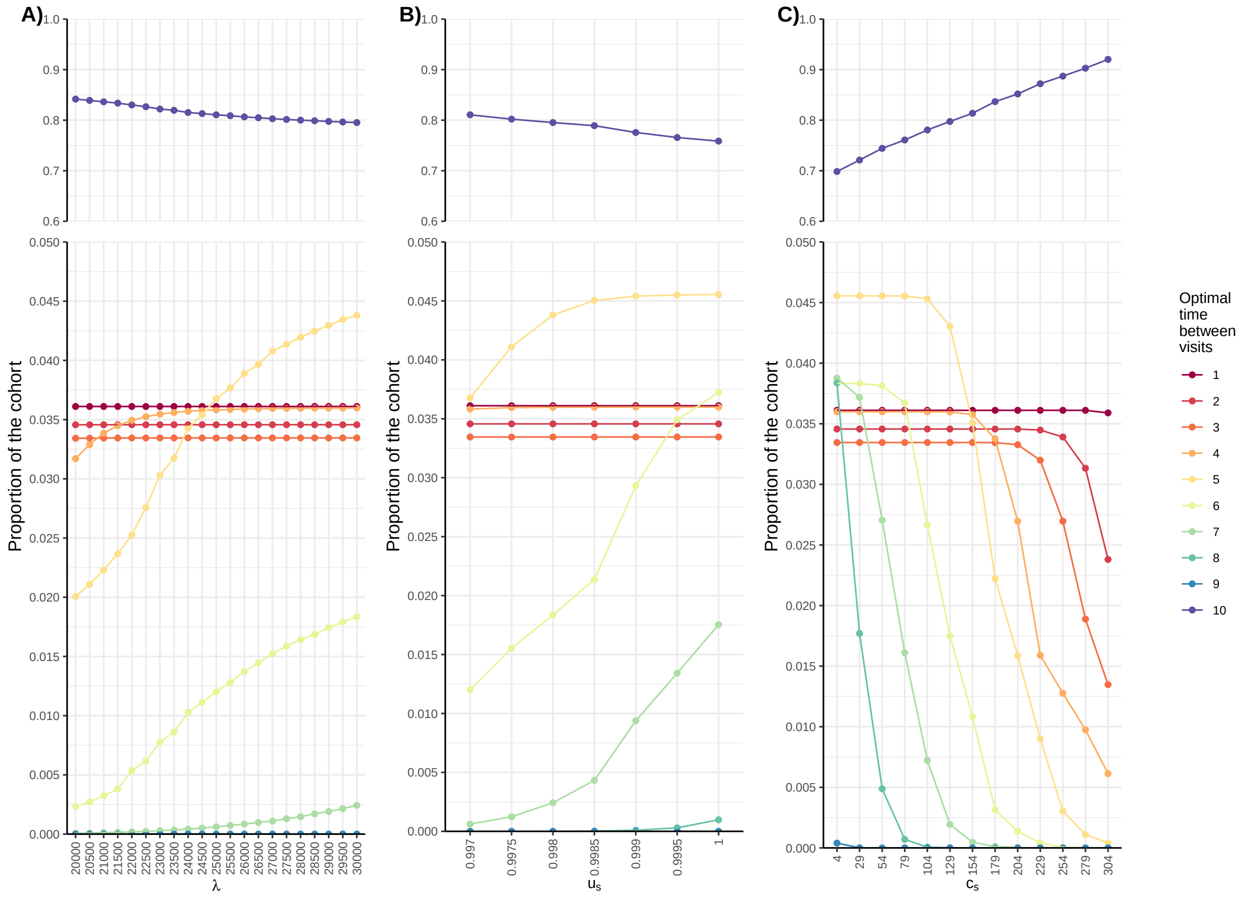

Web Figure 7: Sensitivity analysis of λ (panel A), c_s (panel B), u_s (panel C). In the y-axis of all panels we represent the proportion of the cohort. In panel A, the value of λ is reported in the x-axis and it ranges in $[20,000; 30,000]$ £/year. $u_s = 0.997$, $c_s = 150$ £/year, and $c_\nu = 18.39$ £/visit. In panel B, the value of u_s is reported in the x-axis and it ranges in $[0.997; 1]$. $\lambda = 25,000$ £/year, $c_s = 150$ £/year, and $c_\nu = 18.39$ £/visit. In panel C, the value of c_s is reported in the x-axis and it ranges in $[4; 320]$ £/year. $\lambda = 25,000$ £/year, $u_s = 0.997$, and $c_\nu = 18.39$ £/visit. This figure appears in color in the electronic version of this article.

Web Table 4:
Descriptive characteristics at each landmark age for the women cohorts.

variable	Risk class	40	45	50	55	60	65	70	75	80
AF	Very high risk	0 %	0.27 %	0.45 %	0.65 %	5.5 %	4.37 %	2.8 %	3.4 %	5.63 %
	High risk	0.41 %	0.14 %	0.58 %	0.67 %	0.66 %	0.15 %	0 %	0 %	
	Med-high risk	0.19 %	0.26 %	0.4 %	0.63 %	0.1 %	0 %	0 %	0 %	
	Med-low risk	0.16 %	0.23 %	0.36 %	0.39 %	0 %	0 %			
	Low risk	0.12 %	0.15 %	0.2 %	0.3 %	0 %	0 %			
BP med	Very high risk	95.28 %	91.44 %	92.6 %	83.81 %	80.16 %	77.36 %	57.62 %	52.06 %	62.15 %
	High risk	87.58 %	85.48 %	81.16 %	64.82 %	62.1 %	52.81 %	1.32 %	0.48 %	
	Med-high risk	78.45 %	72.62 %	65.12 %	53.56 %	39.48 %	16.51 %	0 %	0 %	
	Med-low risk	55.12 %	44.92 %	31.13 %	22.49 %	8.83 %	1.68 %			
	Low risk	7.6 %	7.46 %	3.44 %	3.41 %	0.25 %	0 %			
Depression	Very high risk	83.89 %	73.06 %	82.04 %	72.95 %	64.56 %	43.73 %	28.49 %	20.35 %	19.39 %
	High risk	75.57 %	64.06 %	71.49 %	64.54 %	47.25 %	29.01 %	4.37 %	2.18 %	
	Med-high risk	75.16 %	63.31 %	61.84 %	49.22 %	32.29 %	18.48 %	0.7 %	0 %	
	Med-low risk	67.09 %	52.85 %	44.82 %	28.02 %	11.99 %	7.07 %			
	Low risk	26.2 %	25.03 %	14.44 %	7.78 %	1.06 %	0 %			
Diabetes	Very high risk	22.22 %	21.17 %	11.96 %	13.07 %	6.22 %	4.31 %	2.07 %	1.96 %	2.11 %
	High risk	16.15 %	11.42 %	4.81 %	3.5 %	1.4 %	0.37 %	0 %	0 %	
	Med-high risk	6.65 %	5.18 %	2.27 %	1.06 %	0.43 %	0.09 %	0 %	0 %	
	Med-low risk	3.62 %	1.91 %	0.76 %	0.12 %	0.07 %	0 %			
	Low risk	0.3 %	0.15 %	0.06 %	0.01 %	0 %	0 %			
Migraine	Very high risk	63.33 %	52.97 %	41.2 %	26.24 %	20.07 %	15.25 %	7.62 %	6.7 %	5.19 %
	High risk	52.17 %	45.67 %	33.19 %	21.45 %	16.62 %	11.42 %	8.66 %	0.97 %	
	Med-high risk	54.44 %	42.2 %	27.22 %	18.4 %	12.5 %	8.08 %	25.46 %	0 %	
	Med-low risk	42.57 %	30.53 %	16.85 %	11.98 %	6.95 %	3.53 %			
	Low risk	9.99 %	7.99 %	6.1 %	4.98 %	2.73 %	0 %			
Renal dis.	Very high risk	3.06 %	2.61 %	2.93 %	3.14 %	5.04 %	9.28 %	6.86 %	7.86 %	12.18 %
	High risk	1.86 %	1.62 %	2.85 %	2.79 %	3.44 %	3.04 %	0.97 %	0 %	
	Med-high risk	1.46 %	2.17 %	2.17 %	2.08 %	2.14 %	1.17 %	0.09 %	0 %	
	Med-low risk	1.2 %	1.26 %	1.29 %	1.29 %	1.17 %	0.31 %			
	Low risk	0.36 %	0.5 %	0.67 %	0.93 %	1.2 %	0 %			
RA	Very high risk	2.22 %	1.53 %	1.76 %	2.43 %	8.47 %	6.64 %	3.53 %	2.89 %	3.03 %
	High risk	1.04 %	1.06 %	1.59 %	2.03 %	3.28 %	1.95 %	0.03 %	0.48 %	
	Med-high risk	0.7 %	1.58 %	1.68 %	1.82 %	1.53 %	0.44 %	0 %	0 %	
	Med-low risk	0.89 %	1.01 %	1.26 %	1.43 %	0.21 %	0.03 %			
	Low risk	0.66 %	0.86 %	1.05 %	1.43 %	0 %	0 %			
SMI	Very high risk	10 %	6.4 %	9.89 %	8.13 %	5.16 %	4.51 %	1.99 %	1.61 %	1.73 %
	High risk	6.83 %	5 %	5.91 %	3.85 %	2.57 %	0.96 %	0.02 %	0 %	
	Med-high risk	8.3 %	4.24 %	3.92 %	2.42 %	1.31 %	0.23 %	0 %	0 %	
	Med-low risk	5.06 %	2.91 %	1.72 %	0.96 %	0.32 %	0.02 %			
	Low risk	0.78 %	0.8 %	0.23 %	0.2 %	0.03 %	0 %			
SLE	Very high risk	1.11 %	0.9 %	0.45 %	0.41 %	0.43 %	0.43 %	0.3 %	0.32 %	0.23 %
	High risk	0 %	0.35 %	0.37 %	0.46 %	0.37 %	0.32 %	0.2 %	0.24 %	
	Med-high risk	0.51 %	0.45 %	0.41 %	0.38 %	0.28 %	0.22 %	0.18 %	0 %	
	Med-low risk	0.39 %	0.37 %	0.33 %	0.27 %	0.23 %	0.26 %			
	Low risk	0.19 %	0.22 %	0.23 %	0.25 %	0.42 %	0 %			
BMI	Very high risk	1.21 (1.13)	0.89 (1.05)	0.33 (0.86)	0.08 (0.77)	0.01 (0.75)	-0.14 (0.63)	-0.27 (0.53)	-0.37 (0.49)	-0.46 (0.44)
	High risk	0.9 (1.04)	0.58 (0.94)	0.16 (0.78)	-0.04 (0.69)	-0.11 (0.61)	-0.24 (0.52)	-0.45 (0.4)	-0.78 (0.46)	
	Med-high risk	0.54 (0.94)	0.36 (0.86)	0.03 (0.72)	-0.1 (0.62)	-0.21 (0.51)	-0.32 (0.45)	-0.74 (0.4)	-1.18 (0.52)	
	Med-low risk	0.22 (0.83)	0.05 (0.71)	-0.14 (0.6)	-0.21 (0.52)	-0.34 (0.41)	-0.48 (0.42)			
	Low risk	-0.33 (0.48)	-0.32 (0.47)	-0.34 (0.46)	-0.39 (0.44)	-0.66 (0.39)	-0.93 (0.34)			
HDL	Very high risk	-1.09 (0.44)	-0.7 (0.57)	-0.46 (0.57)	-0.32 (0.57)	-0.03 (0.61)	0.01 (0.59)	0.2 (0.59)	0.29 (0.59)	0.3 (0.58)
	High risk	-0.87 (0.48)	-0.57 (0.53)	-0.28 (0.55)	-0.14 (0.52)	0.09 (0.56)	0.19 (0.54)	0.44 (0.56)	1.77 (0.67)	
	Med-high risk	-0.68 (0.47)	-0.43 (0.54)	-0.16 (0.53)	-0.01 (0.49)	0.19 (0.52)	0.31 (0.52)	1.11 (0.82)	2.52 (0.28)	
	Med-low risk	-0.48 (0.42)	-0.26 (0.46)	-0.02 (0.47)	0.17 (0.45)	0.33 (0.51)	0.57 (0.65)			
	Low risk	-0.12 (0.33)	0.01 (0.4)	0.2 (0.46)	0.46 (0.57)	0.71 (0.71)	1.79 (0.83)			
SBP	Very high risk	0.19 (0.65)	0.25 (0.63)	0.06 (0.6)	0.07 (0.55)	0.17 (0.51)	0.21 (0.46)	0.23 (0.39)	0.29 (0.39)	0.35 (0.42)
	High risk	0.02 (0.58)	0.03 (0.58)	-0.08 (0.54)	-0.04 (0.5)	0.04 (0.43)	0.08 (0.37)	0.04 (0.3)	0 (0.36)	
	Med-high risk	-0.23 (0.55)	-0.16 (0.53)	-0.2 (0.51)	-0.12 (0.46)	-0.05 (0.37)	0.01 (0.33)	-0.34 (0.32)	-0.3 (0.25)	
	Med-low risk	-0.45 (0.47)	-0.38 (0.45)	-0.35 (0.42)	-0.24 (0.38)	-0.18 (0.34)	-0.18 (0.34)			
	Low risk	-0.84 (0.33)	-0.68 (0.34)	-0.53 (0.36)	-0.42 (0.36)	-0.57 (0.33)	-0.83 (0.23)			
Smoke	Very high risk	0.71 (0.24)	0.72 (0.24)	0.68 (0.26)	0.72 (0.24)	0.51 (0.3)	0.4 (0.29)	0.26 (0.23)	0.2 (0.19)	0.18 (0.19)
	High risk	0.66 (0.26)	0.68 (0.24)	0.6 (0.26)	0.61 (0.25)	0.41 (0.26)	0.29 (0.22)	0.17 (0.1)	0.08 (0.08)	
	Med-high risk	0.64 (0.24)	0.64 (0.24)	0.56 (0.26)	0.51 (0.25)	0.35 (0.21)	0.24 (0.14)	0.07 (0.08)	0.07 (0.08)	
	Med-low risk	0.6 (0.24)	0.6 (0.24)	0.5 (0.24)	0.38 (0.18)	0.28 (0.15)	0.18 (0.12)			
	Low risk	0.46 (0.2)	0.43 (0.2)	0.38 (0.18)	0.27 (0.16)	0.14 (0.13)	0.04 (0.06)			
TCHOL	Very high risk	0.08 (0.56)	0.35 (0.68)	0.31 (0.6)	0.42 (0.6)	0.44 (0.53)	0.43 (0.51)	0.39 (0.46)	0.36 (0.46)	0.3 (0.48)
	High risk	0 (0.56)	0.15 (0.57)	0.22 (0.51)	0.38 (0.5)	0.43 (0.44)	0.44 (0.43)	0.44 (0.4)	0.31 (0.54)	
	Med-high risk	-0.09 (0.49)	0.09 (0.5)	0.15 (0.44)	0.33 (0.42)	0.42 (0.4)	0.43 (0.38)	0.43 (0.48)	0.12 (0.38)	
	Med-low risk	-0.22 (0.4)	-0.05 (0.39)	0.09 (0.38)	0.29 (0.36)	0.39 (0.35)	0.39 (0.39)			
	Low risk	-0.38 (0.24)	-0.22 (0.28)	0.01 (0.31)	0.22 (0.35)	0.24 (0.41)	0.02 (0.61)			
Townsend 20	Very high risk	15.69 (3.62)	14.98 (4.16)	14.74 (4.17)	13.65 (4.72)	12.97 (4.81)	11.66 (5.05)	9.44 (5.19)	8.69 (5.19)	9.01 (5.2)
	High risk	15.36 (3.75)	14.41 (4.43)	13.27 (4.81)	12.69 (4.97)	11.32 (5.1)	9.23 (5.17)	5.59 (3.8)	4.22 (2.96)	
	Med-high risk	15.04 (4.18)	13.9 (4.68)	12.4 (5.16)	11.28 (5.31)	9.59 (5.12)	7.61 (4.41)	3.68 (2.67)	2 (1.28)	
	Med-low risk	14.1 (4.58)	12.93 (5.09)	11.04 (5.4)	9.18 (5.06)	6.32 (4.13)	4.27 (3.12)			
	Low risk	9.02 (5.52)	8.31 (5.31)	7.04 (4.75)	5.39 (3.97)	3.14 (2.27)	2.5 (1.64)			

Web Table 5:

Descriptive characteristics at each landmark age for the men cohorts.

variable	Risk class	40	45	50	55	60	65	70	75	80
AF	Very high risk	1.17 %	0.81 %	1.02 %	1.39 %	2.42 %	2.35 %	3.42 %	5.45 %	8.27 %
	High risk	0.6 %	1.1 %	0.95 %	0.82 %	0.01 %	0 %	0 %		
	Med-high risk	1.01 %	0.71 %	0.54 %	0.62 %	0.01 %	0 %			
	Med-low risk	0.44 %	0.33 %	0.41 %	0.69 %	0 %	0 %			
	Low risk	0.2 %	0.29 %	0.32 %	0.67 %	0 %				
BP med	Very high risk	84.49 %	80.29 %	59.73 %	50.49 %	36.45 %	31.51 %	36.19 %	44.84 %	55.08 %
	High risk	78.49 %	56.2 %	30.34 %	11.87 %	5.43 %	2.38 %	0 %		
	Med-high risk	60.98 %	29.73 %	9.63 %	3.18 %	3.89 %	1.39 %			
	Med-low risk	18.14 %	4.46 %	2.22 %	2.15 %	2.56 %	0 %			
	Low risk	1.15 %	0.85 %	1.1 %	0 %	0 %				
Depression	Very high risk	56.32 %	44.93 %	39.64 %	31.72 %	21.13 %	14.41 %	11.64 %	10.35 %	9.81 %
	High risk	54.67 %	39.5 %	26.99 %	14.84 %	4.48 %	2.91 %	4 %		
	Med-high risk	48.83 %	32.99 %	17.45 %	4.89 %	3.11 %	2.08 %			
	Med-low risk	42.19 %	15.15 %	6.75 %	3.68 %	1.87 %	0 %			
	Low risk	7.22 %	5.4 %	3.78 %	2.68 %	0 %				
Diabetes	Very high risk	24.51 %	4.42 %	6.79 %	4.17 %	2.92 %	2.45 %	2.94 %	3.7 %	3.82 %
	High risk	8.84 %	3.35 %	1.7 %	0.78 %	0.16 %	0.28 %	0 %		
	Med-high risk	5.5 %	1.8 %	0.61 %	0.29 %	0.09 %	1.16 %			
	Med-low risk	1.11 %	0.58 %	0.16 %	0.18 %	0.51 %	0 %			
	Low risk	0.09 %	0.46 %	0.18 %	1.34 %	0 %				
Migraine	Very high risk	8.6 %	17.17 %	10.67 %	7.61 %	4.75 %	3.45 %	3.16 %	2.63 %	2.27 %
	High risk	10.55 %	15.2 %	6.35 %	4.13 %	2.2 %	3.4 %	0 %		
	Med-high risk	9.19 %	10.16 %	4.5 %	1.58 %	1.97 %	2.31 %			
	Med-low risk	6.18 %	3.65 %	1.87 %	1.04 %	2.04 %	0 %			
	Low risk	3.84 %	1.07 %	1.3 %	0 %	0 %				
Renal dis.	Very high risk	3.52 %	1.92 %	1.91 %	1.75 %	2.19 %	2.26 %	3.66 %	6.29 %	10.26 %
	High risk	1.71 %	1.94 %	1.38 %	0.86 %	0.28 %	2.73 %	0 %		
	Med-high risk	1.5 %	1.03 %	0.64 %	0.62 %	0.19 %	3.01 %			
	Med-low risk	0.68 %	0.42 %	0.5 %	0.96 %	0.34 %	50 %			
	Low risk	0.25 %	0.39 %	0.83 %	0 %	0 %				
RA	Very high risk	0.65 %	0.72 %	0.78 %	0.78 %	1.3 %	1.09 %	1.24 %	1.38 %	1.44 %
	High risk	0.5 %	0.55 %	0.67 %	0.6 %	0.03 %	0.07 %	0 %		
	Med-high risk	0.55 %	0.55 %	0.49 %	0.53 %	0.04 %	0 %			
	Med-low risk	0.38 %	0.41 %	0.47 %	0.9 %	0 %	0 %			
	Low risk	0.31 %	0.42 %	0.72 %	0.67 %	0 %				
SMI	Very high risk	6.65 %	5.95 %	3.73 %	2.31 %	1.92 %	1.22 %	1.2 %	1.14 %	1.11 %
	High risk	4.42 %	5.52 %	2.16 %	1.16 %	0.14 %	0.14 %	0 %		
	Med-high risk	3.62 %	3.39 %	1.33 %	0.61 %	0.07 %	0.23 %			
	Med-low risk	2.74 %	1.16 %	0.63 %	0.75 %	0.17 %	0 %			
	Low risk	1.17 %	0.49 %	0.74 %	0.67 %	0 %				
SLE	Very high risk	0 %	0.1 %	0.09 %	0.09 %	0.07 %	0.08 %	0.09 %	0.09 %	0.09 %
	High risk	0.2 %	0.02 %	0.11 %	0.05 %	0.04 %	0.14 %	0 %		
	Med-high risk	0 %	0.09 %	0.05 %	0.06 %	0.07 %	0.23 %			
	Med-low risk	0.05 %	0.05 %	0.04 %	0.08 %	0 %	0 %			
	Low risk	0.04 %	0.02 %	0.07 %	0 %	0 %				
BMI	Very high risk	0.77 (1.09)	0.45 (0.95)	0.09 (0.73)	-0.1 (0.62)	-0.22 (0.53)	-0.31 (0.49)	-0.4 (0.49)	-0.46 (0.47)	-0.56 (0.44)
	High risk	0.39 (0.9)	0.16 (0.69)	-0.14 (0.56)	-0.22 (0.44)	-0.32 (0.37)	-0.62 (0.44)	-1.16 (0.51)		
	Med-high risk	0.21 (0.77)	-0.05 (0.57)	-0.18 (0.44)	-0.26 (0.39)	-0.5 (0.45)	-0.89 (0.44)			
	Med-low risk	-0.11 (0.53)	-0.19 (0.37)	-0.25 (0.38)	-0.49 (0.47)	-0.82 (0.43)	-1.02 (0.78)			
	Low risk	-0.28 (0.32)	-0.33 (0.38)	-0.52 (0.45)	-0.94 (0.44)	-1.48 (0.18)				
HDL	Very high risk	-0.7 (0.51)	-0.41 (0.52)	-0.23 (0.51)	-0.07 (0.5)	0.08 (0.5)	0.2 (0.56)	0.28 (0.63)	0.28 (0.64)	0.3 (0.62)
	High risk	-0.49 (0.46)	-0.28 (0.49)	-0.07 (0.43)	0.08 (0.41)	0.25 (0.46)	0.78 (0.81)	1.96 (1.31)		
	Med-high risk	-0.39 (0.44)	-0.18 (0.41)	-0.01 (0.37)	0.17 (0.43)	0.63 (0.76)	1.78 (1.08)			
	Med-low risk	-0.22 (0.32)	-0.07 (0.3)	0.1 (0.41)	0.68 (0.81)	1.75 (1.02)	2.53 (0.98)			
	Low risk	-0.07 (0.31)	0.11 (0.48)	0.65 (0.83)	2.34 (0.93)	4.04 (0.08)				
SBP	Very high risk	0.2 (0.64)	0.16 (0.58)	-0.06 (0.54)	-0.06 (0.49)	-0.02 (0.44)	-0.01 (0.41)	0.01 (0.44)	0.06 (0.45)	0.07 (0.46)
	High risk	-0.06 (0.55)	-0.13 (0.46)	-0.26 (0.41)	-0.19 (0.34)	-0.15 (0.33)	-0.4 (0.37)	-0.6 (0.37)		
	Med-high risk	-0.21 (0.49)	-0.3 (0.38)	-0.32 (0.33)	-0.28 (0.32)	-0.37 (0.39)	-0.59 (0.43)			
	Med-low risk	-0.44 (0.34)	-0.43 (0.27)	-0.4 (0.31)	-0.48 (0.37)	-0.55 (0.43)	-0.54 (0.38)			
	Low risk	-0.57 (0.25)	-0.57 (0.29)	-0.61 (0.35)	-0.66 (0.37)	-0.59 (0.56)				
Smoke	Very high risk	0.7 (0.22)	0.68 (0.23)	0.65 (0.25)	0.54 (0.28)	0.4 (0.26)	0.29 (0.24)	0.22 (0.23)	0.17 (0.22)	0.14 (0.21)
	High risk	0.68 (0.22)	0.64 (0.23)	0.6 (0.24)	0.42 (0.2)	0.28 (0.15)	0.15 (0.15)	0.06 (0.08)		
	Med-high risk	0.65 (0.21)	0.65 (0.23)	0.5 (0.19)	0.34 (0.17)	0.17 (0.16)	0.12 (0.12)			
	Med-low risk	0.66 (0.19)	0.54 (0.17)	0.39 (0.17)	0.2 (0.19)	0.14 (0.15)	0.04 (0.02)			
	Low risk	0.52 (0.18)	0.38 (0.2)	0.18 (0.18)	0.15 (0.15)	0.13 (0.15)				
TCHOL	Very high risk	0.68 (0.75)	0.59 (0.59)	0.52 (0.52)	0.45 (0.48)	0.39 (0.42)	0.3 (0.41)	0.19 (0.43)	0.08 (0.44)	0.02 (0.45)
	High risk	0.51 (0.57)	0.5 (0.48)	0.44 (0.42)	0.41 (0.36)	0.36 (0.32)	0.16 (0.46)	-0.44 (0.66)		
	Med-high risk	0.47 (0.5)	0.43 (0.4)	0.42 (0.33)	0.37 (0.33)	0.23 (0.44)	0.03 (0.57)			
	Med-low risk	0.34 (0.33)	0.37 (0.28)	0.36 (0.32)	0.22 (0.46)	0.03 (0.53)	-1.1 (0.51)			
	Low risk	0.24 (0.24)	0.27 (0.33)	0.17 (0.45)	0.02 (0.6)	0.09 (0.25)				
Townsend 20	Very high risk	13.97 (4.8)	12.87 (5.15)	12.81 (5.16)	11.26 (5.35)	10.34 (5.25)	8.7 (5.23)	8.39 (5.17)	8.53 (5.2)	8.73 (5.21)
	High risk	13.62 (4.75)	12.21 (5.39)	11.63 (5.33)	10.21 (5.31)	5.96 (4.02)	4.83 (3.7)	3.2 (2.45)		
	Med-high risk	12.92 (5.18)	12.19 (5.36)	10.91 (5.25)	6.42 (4.42)	4.99 (3.87)	4.43 (3.54)			
	Med-low risk	12.89 (5.09)	10.29 (5.16)	6.48 (4.45)	5.36 (4.12)	4.54 (3.61)	6.5 (0.71)			
	Low risk	8.65 (5.41)	5.98 (4.81)	4.95 (3.99)	4.72 (4.23)	5 (1.73)				
